# Lipidomic Profiling Reveals Disruption of Lipid Metabolism in Valproic Acid-Induced Hepatotoxicity

**DOI:** 10.3389/fphar.2019.00819

**Published:** 2019-07-19

**Authors:** Shansen Xu, Yanan Chen, Yiyi Ma, Ting Liu, Mingming Zhao, Zhanyou Wang, Limei Zhao

**Affiliations:** ^1^Department of Pharmacy, Shengjing Hospital of China Medical University, Shenyang, China; ^2^Shanghai AB Sciex Analytical Instrument Trading Co. Ltd., Shanghai, China; ^3^Institute of Health Sciences, Key Laboratory of Medical Cell Biology of Ministry of Education, China Medical University, Shenyang, China

**Keywords:** valproic acid, nontargeted lipidomics, lipid metabolism genes, hepatotoxicity, Akt–PPARγ pathway

## Abstract

Valproic acid (VPA) is one of the most widely prescribed antiepileptic drugs, as VPA-induced hepatotoxicity is one of the most severe adverse reaction that can lead to death. The objective of this study was to gain an understanding of dysregulated lipid metabolism in mechanism of hepatotoxicity. Nontargeted lipidomics analysis with liquid chromatography–quadrupole-time-of-flight mass spectrometry (LC-Q-TOF/MS) was performed to explore differential lipids from the patient serum and L02 cells. Lipidomics data interpretation was augmented by gene expression analyses for the key enzymes in lipid metabolism pathways. From patient serum lipidomics, pronouncedly changed lipid species between abnormal liver function (ALF) patients and normal liver function (NLF) patients were identified. Among these lipid species, LPCs, Cers, and SMs were markedly reduced in the ALF group and showed negative relationships with liver injury severity [alanine aminotransferase (ALT) levels], while significantly increased triacylglycerols (TAG) with higher summed carbon numbers demonstrated a positive relationship with ALT levels. Regarding lipidomics in hepatic L02 cells, TAG was markedly elevated after VPA exposure, especially in TAGs with more than 53 summed carbons. Besides, gene expression analysis revealed dysregulated lipid metabolism in VPA-treated L02 cells. Peroxime proliferators-activated receptor (PPARγ) pathway played an important role in VPA-induced lipid disruption through inducing long-chain fatty acid uptake and TAG synthesis, which was also regulated by Akt pathway. Our findings present that VPA-induced lipid metabolism disruption might lead to lipotoxicity in the liver. This approach is expected to be applicable for other drug-induced toxicity assessments.

## Introduction

Valproic acid (VPA) is an antiepileptic drug (AED) that is widely used in the treatment for epileptic children and is effective against many types of seizure disorders either alone or as a component of a multidrug regimen (Bűdi et al., 2015). However, VPA has several side effects, such as weight gain, blood dyscrasias, and liver injury (Nanau and Neuman, 2013). VPA-induced hepatotoxicity is a fatal and idiosyncratic adverse drug reaction; the hepatotoxicity incidence in children is about 1/5,000, and 1/500 in a high-risk population (Dreifuss et al., 1987). Hepatitis-like syndrome, hyperammonemia, and nonalcoholic fatty liver disease (NAFLD) are considered major symptoms of VPA-induced hepatotoxicity in clinic (Verrotti et al., 2009; Verrotti et al., 2011; Farinelli, 2015). Previous studies had revealed that the hepatotoxic metabolites of VPA played a critical role in its pathogenesis (Park et al., 2005; Walgren et al., 2005).

Recent studies focused on the role of VPA in NAFLD development, and the results revealed that NAFLD occurrence is significantly higher in VPA-treated patients than in those treated with other AEDs (Luef et al., 2004; Luef et al., 2009; Verrotti et al., 2011; Saleh et al., 2012). Thus, it is critical to identify noninvasive biomarkers (indicators of adverse effects) for the early diagnosis of liver injury induced by VPA prior to the occurrence of irreversible damage. Huo et al. (2014) indicated that VPA-induced hepatotoxicity disrupted glycolysis, lipid metabolism, and amino acid metabolism in humans through metabolomic approach. A metabolic profiling study revealed that VPA also altered organic acid metabolism in pediatric urine by dysregulating branched-chain amino acid metabolism and oxidative stress (Price et al., 2011). Moreover, several metabolomic studies found disrupted glycine in serum and hippuric acid in rat urine, which were considered as VPA-induced hepatotoxicity biomarkers (Lee et al., 2009; Sun et al., 2010; Zhang et al., 2014).

Nowadays, lipidomics has become a hot area in disease research, as lipids play diverse roles in cellular functions. Altered lipid profiles correlated with disease progression will provide new insights to deeply understand the pathogenic mechanisms of chemical-induced toxicities in disease (Dobrosotskaya, 2002; Maceyka and Spiegel, 2014; Yang et al., 2016). Thus, lipidomics may provide a chance for the diagnosis of diseases in the early stage and provide the possibility for successful treatment. Although there is one metabolomics study about VPA-induced hepatotoxicity (Huo et al., 2014), human pediatric lipidomics provides a new approach to investigating the mechanistic basis to VPA-induced hepatotoxicity.

In this study, a nontargeted lipidomics approach based on liquid chromatography–quadrupole-time-of-flight mass spectrometry (LC-Q-TOF/MS) was applied to explore the candidate biomarkers for VPA-induced hepatotoxicity. To investigate the possible mechanism of VPA-induced dysregulation of lipid metabolism, relative gene expression, including fatty acid uptake, lipid transport, and TAG synthesis, was tested in hepatic cell line L02. We found that the Akt–PPARγ pathway participated in VPA-induced lipid disruption, which acts as a critical pathway in the regulation of adipose differentiation, lipid storage, and genes involved in energy storage and utilization (Rogue et al., 2010).

## Materials and Methods

### Reagents

Liquid-chromatography-grade acetonitrile, methanol, dichloromethane, and isopropanol were purchased from Fisher Scientific (Pittsburgh, PA, USA). Internal standard TAG 45:0, sodium valproate, Oil Red O powders, and ammonium acetate were purchased from Sigma-Aldrich (St. Louis, MO, USA). GW9662, rosiglitazone, and LY294002 were purchased from MedChemExpress (Shanghai, China). Ultrapure water was prepared using a Milli-Q water purification system procured from Millipore Corp. (Billerica, MA, USA).

### Lipidomic Profiling in Patient Serum

#### Sample Collection and Clinical Information

A total of 23 epileptic children (aged 6 months to 14 years) from the Department of Pediatrics at Shengjing Hospital of China Medical University were enrolled. This study was approved by the ethics committee of Shengjing Hospital and written informed consent was obtained from the guardians of each pediatric patient. All patients were diagnosed with symptomatic epilepsy and had been treated with valproate sodium for at least 2 months. Comprehensive demographic details were collected from patients at the time of enrollment in the study, including age, weight, sex, concurrent medications, duration of current therapy, and compliance details (as shown in **Table 1**). Additionally, liver and renal function test results were recorded including albumin, alanine aminotransferase (ALT), aspartate aminotransferase (AST), alkaline phosphatase (ALP), γ-glutamyl-transferase (GGT), total bilirubin (TBiL), blood urea nitrogen (BUN), and serum creatinine (Scr). According to the levels of the aforementioned liver function indicators, patients were divided into two groups. Those patients with all indicator levels lower than the upper reference limit were placed into the non-abnormal liver function group (NLF), and those with any indicator level exceeding 2× the upper limit of normal (ULN) were placed into the abnormal liver function (ALF) group. Plasma samples were stored at −80°C until additional extraction and analysis were performed.

**Table 1:** Clinical biochemical information of the patients enrolled in this study.

Parameters	NLF subjects		ALF subjects		*P*
	Mean ± SD	Range	Mean ± SD	Range	
Gender (male/female)	5/5	−	8/5	−	−
Age (years)	7.3 ± 4.2	2–13	7.0 ± 5.0	0.5–14	0.895
Weight (kg)	32.4 ± 16.0	13–65	39.5 ± 25.4	9–85	0.449
BMI	18.3 ± 4.0	13.5–25.4	21.5 ± 4.7	15.0–28.4	0.102
Daily dose (mg/kg)	15.7 ± 5.1	7.7–23.8	14.0 ± 6.1	5.3–25.0	0.532
VPA concentration (µg/mL)	47.67 ± 26.85	11.33–82.32	57.52 ± 29.90	15.43–102.85	0.423
Albumin (g/L)	44.3 ± 3.4	38–49	45.4 ± 3.6	38–52	0.487
ALT (U/L)	11.3 ± 2.8	8–15	128.4 ± 57.1	88–251	<0.001
AST (U/L)	21.4 ± 5.7	13–31	98.1 ± 68.8	39–298	0.002
ALP (U/L)	182.8 ± 35.1	145–229	244.5 ± 76.7	147–390	0.048
GGT (U/L)	12.1 ± 2.6	9–17	37.5 ± 25.0	13–90	0.010
TBiL (µmol/L)	7.32 ± 2.18	4.2–12.1	7.09 ± 3.11	3.0–13.4	0.841
BUN (mmol/L)	4.07 ± 0.86	2.9–5.4	4.27 ± 1.36	1.9–7.0	0.690
Scr (µmol/L)	32.5 ± 9.5	18.2–51.6	38.9 ± 12.4	15.6–56.2	0.194

Reference ranges: Albumin, 35–53 g/L; ALT, 0–40 U/L; AST, 5–34 U/L; ALP, 40–375 U/L; GGT, 9–64 U/L; BUN, 3–9.2 mmol/L; TBIL, 3.4–20.5 µmol/L; SCr, 45–84 µmol/L; VPA, 50–100 µg/ml.

#### Preparation of Serum Lipid Extraction for LC-MS Analysis

For serum lipidomic analysis, 50 μl of experimental plasma samples was aliquoted into glass culture tubes, followed by dilution with 1.9 ml of methanol:dichloromethane:water (20:9:9, *v/v/v*) with internal standard TAG 45:0 (0.2 μg/ml). Samples were vortexed for 5 s and then allowed to stand for 30 min at room temperature. An additional 1.9 ml of dichloromethane/water (9:10, *v/v*) was added to extracts, and extracts were gently vortexed for 5 s, and then centrifuged at 3,000 rpm for 10 min at 4°C. The bottom organic layer was transferred to a new test tube for each extract. Another 1.8 ml of dichloromethane was added to the original extract test tube. Original extracts were gently vortexed and centrifuged. The bottom layers were taken again and added to the previous aliquots. The combined bottom layers were concentrated under nitrogen and reconstituted in 250 μl of running solution (methanol/dichloromethane; 1:1, *v/v*) in 10 mM ammonium acetate. Extracts were transferred to inserts and placed in vials for LC-MS analysis. Notably, pooled quality-control (QC) samples that consisted of equal volumes of plasma from each sample were pretreated in the same way as the individual biological samples. These QC samples were used to appraise the reliability of the entire experiment including sample preparation and LC-MS sequence runs. One QC sample was inserted into the analysis sequence every four real samples.

#### LC-MS Analysis of Serum Lipid Extract

An untargeted lipidomic analysis of plasma samples was performed using a SCIEX ExionLC system coupled with SCIEX Triple TOF 5600 System (SCIEX, Framingham, MA, USA). Chromatographic separation was performed on a Kinetex C18 (2.1 mm × 100 mm; 2.6 μm) column with the temperature set at 40°C and a flow rate of 0.40 ml/min. The mobile phase was composed of solvent A [acetonitrile:methanol:water (1:1:2, *v/v/v*) in 5 mM ammonium acetate] and solvent B [isopropanol:methanol (2:1, *v/v*) in 5 mM ammonium acetate]. The gradient elution was initially started from 30% B, increased to 40% at 2 min, increased further to 95% at 12 min, held for the next 3 min, then reduced to 30% at 15.1 min, and finally maintained for 2.9 min. The volume of sample injected into the column was 2 μl.

Mass spectrometry was operated with the following parameters: ion spray voltage, 5.5 kV (+) and 5.5 kV (−); curtain gas, 35 PSI; declustering potential, 80 V (+) and 80 V (−); collision energy, 35 V (+) and 35 V (−); and interface heater temperature, 550°C. Collision energy spread was set at 15 for both positive and negative modes, along with dynamic background subtraction (DBS). Experiments were run with 250 ms accumulation time for TOF MS (*m/z* 350–1100) and 50 ms accumulation time for TOF MS/MS (*m/z* 100–1100) combined with IDA mode.

#### Data Processing and Differential Lipids Identification

LipidView software (version 1.2; SCIEX, Framingham, MA, USA) was first used to filter candidate lipids, and lipid identification was based on exact mass, retention time, and MS/MS pattern. PeakView workstation (version 2.2; SCIEX, Framingham, MA, USA) was used to check lipid MS/MS information, and MultiQuant software (version 3.0; SCIEX, Framingham, MA, USA) was employed to obtain lipid peak area. MarkerView (version 1.3; SCIEX, Framingham, MA, USA) was performed to analyze data differences. Subsequent data analysis was performed based on a peak table consisting of identified lipid species from positive and negative ion modes.

Principal component analysis (PCA), partial least squares discriminate analysis (PLS-DA), and orthogonal projection to latent structures discrimination analysis (OPLS-DA) with unit variance (UV) scaling were performed using SIMCA-P software (version 13.0; Umetrics, Umea, Sweden). Variable importance in projection (VIP) values were used to select biomarkers from the PLS-DA model. Variables with a VIP exceeding 1 showed a higher than average influence on classification. The calculated R2Y (cum.) estimates the goodness of fit of the model that represents the fraction of explained Y-variation, whereas Q2 (cum.) estimates prediction ability. Differential metabolite selection was based on fold change greater than 1.5, and a *P* value less than 0.05 was considered to indicate statistical significance.

### Lipidomics Analysis and Lipid Metabolism Gene Expressions in L02 Cells

#### Cell Culture and Treatment

The human normal hepatic cell line L02 (Shanghai Institute of Biological Sciences, Chinese Academy of Science, Shanghai, China) was maintained in DMEM containing 10% FBS (Hyclone, Logan, UT, USA) and 1% penicillin/streptomycin (Hyclone, Logan, UT, USA) in a 5% CO_2_ humidified 37°C incubator. Fresh medium was added to cells every 2–3 days.

#### Cytotoxicity Assay and Biochemical Analysis

Briefly, 8 × 10^3^ exponentially growing cells per well were seeded in 96-well plates and allowed to adhere overnight before treatment. Cells were then exposed to various concentrations of VPA (0, 0.5, 1, 5, or 10 mM) in a final volume of 100 μl of DMEM with 10% FBS. After 24-h treatment, 20 μl of MTT reagent (5 mg/ml) was added to each well, and cells were further incubated at 37°C for 4 h. Next, MTT solution was removed and 150 μl of DMSO was added to each well. To determine cell viability, a scanning multi-well spectrophotometer (BioTek, Winooski, VT, USA) was used to read OD values at 570 nm. The variation in metabolic activity shows a good correlation with the decline in cell growth.

ALT and AST activities were assayed as markers of hepatotoxicity. L02 cells were seeded in six-well plates at a density of 1 × 10^6^ cells/well overnight. New culture medium was added to wells containing different concentrations of drug. After 24 h, the culture medium was collected to determine ALT and AST activities using an assay kit (Nanjing Jiancheng Biology Engineering, Nanjing, China). All operations followed the kit instructions.

The activity of lactate dehydrogenase (LDH) releasing, as an index of hepatotoxicity, was also evaluated in the present study. The LDH release activity was determined using an LDH cytotoxicity assay kit (Beyotime Biotechnology, Beijing, China). L02 cells (1 × 10^4^ cells) were cultured in 96-well plates, and after different concentrations of VPA treatment for 24 h in DMEM, LDH activity was measured in medium according to the manufacturer’s instructions.

#### Cell Lipid Extraction and LC-MS Analysis

Cells (2 × 10^6^) were treated with different concentrations of VPA (0, 1, or 5 mM) for 24 h in 60-mm cell culture dishes and were designated control, low-dose VPA (VL, 1 mM), and high-dose VPA (VH, 5 mM) groups, respectively. Then, supernatants were removed and dishes were washed with PBS three times. Next, scraped cells were disrupted with 0.9 ml of water under ultrasonic condition in ice (30 s). Lipid extraction and lipidomic profiling of cells followed the same protocol as that used for serum lipid extraction and LC-MS analysis, except that TOF MS *m/z* ranged from 200 to 1100.

#### Oil Red O Staining and TAG Assays

Oil Red O Stock solution (0.5%) was prepared in isopropyl alcohol. L02 cells were seeded in six-well plates and were treated with different concentrations of VPA for 24 h. Then, the cells were washed twice with PBS and fixed with 4% paraformaldehyde for 20 min, followed by staining with Oil Red O in 60% isopropyl alcohol for 30 min. Then, cell images were performed immediately under a bright-field microscope (Olympus, Tokyo, Japan) at 400× magnification.

The total TAG content was determined by an assay kit (Nanjing Jiancheng Biology Engineering, Nanjing, China). Total protein in cells was determined using the BCA protein assay kit (Beyotime Biotechnology, Beijing, China) and used for normalization purposes. All of the operations followed the kit instructions.

#### Quantitative Real-Time PCR Analysis of Lipid Metabolism Genes

Total RNA was prepared from VPA (1 mM)-treated L02 cells using the simple RNA Extract kit (Tiangen Biotech, Beijing, China) and cDNA was generated from 1 μg of RNA with a GoScript Reverse Transcriptase kit (Promega, Madison, WI, USA). Quantitative PCR (qPCR) was performed using GoTaq qPCR Master Mix (Promega, Madison, WI, USA) and an ABI Prism 7500 system (Applied Biosystems, Foster City, CA, USA). Melting curve analysis was performed to confirm the production of a single product in each reaction. Primers used for gene expression studies are listed in **Supplementary Table S1**. Changes in gene expression were determined by normalizing mRNA levels to those of GAPDH as an internal control and fold change was calculated using the 2^−ΔΔCt^ method.

#### Western Blot Analysis

After treatment, the medium was removed and cells were washed twice with ice-cold PBS. The cells were scraped and lysed in RIPA buffer (Beyotime Biotechnology, Beijing, China) with protease inhibitors (MedChemExpress, Shanghai, China), and the protein concentration was measured using the BCA protein assay kit (Beyotime Biotechnology, Beijing, China) using BSA as the standard. For Western blotting analysis, prepared proteins (30–60 μg) were performed by standard SDS-PAGE with 10% (w/v) polyacrylamide gels and transferred onto the polyvinylidene fluoride (PVDF) membranes (Millipore, Bedford, Massachusetts, USA). Then, the membranes were incubated in blocking solution (5% nonfat milk in TBS with 0.1% Tween 20) for 1 h at room temperature and incubated overnight at 4°C with anti-DGAT2, CD36, L-FABP (from Santa Cruz Biotechnology, California, USA), ChREBP, SREBP-1c (from Abcam, Cambridge, Massachusetts, USA), CPT1, FAS, ACC1, PPARγ, PI3K, Akt, p-Akt (Ser473), and GAPDH (from Cell Signaling Technology, Beverly, Massachusetts, USA). Next, the membranes were washed three times with TBS containing 0.1% Tween 20 for 5 min with shaking and then incubated with appropriate peroxidase-conjugated secondary antibodies (Cell Signaling Technology, Beverly, Massachusetts, USA) for 2 h at room temperature and washed three times for 5 min. Immunodetection analysis was accomplished using an enhanced chemiluminescence solution (ECL, Millipore, Bedford, MA, USA). Band densities were quantified using ImageJ Software. The relative expression levels of purpose proteins were normalized to GAPDH levels.

### Statistical Analysis

Statistical analyses were conducted using Prism 7 (GraphPad Prism, La Jolla, CA, USA). All values are expressed as means ± SD (standard deviations). One-way ANOVA was performed to determine significant differences between control and VPA-treated cells, followed by Dunnett’s multiple comparison test. Receiver operating characteristic (ROC) analysis was figured out by SPSS software 22.0 (SPSS Inc., Chicago, USA). A *P* value < 0.05 was considered statistically significant.

## Results

### Patient Serum Lipid Profiling

#### Clinical Characteristics of Patients

As shown in **Table 1**, a total of 23 epileptic children administered VPA were enrolled in this study. According to liver function test results, 10 patients were assigned to the NLF group, and 13 patients with ALF were assigned to the ALF group. No significant differences in age, sex, daily VPA dose, or VPA trough serum concentration were found between the NLF and ALF patients. All patients enrolled in the ALF group were diagnosed with ALF according to ALT and AST levels (ALT > 2 × ULN and/or AST > 2 × ULN), which are considered good indicators of liver cell damage; ALT is considered as more specific for liver injury than AST. Serum ALT, AST, ALP, and GGT activity were significantly higher in the ALF group than those in the NLF group (*P* < 0.05). Other clinical parameters showed no differences among patient groups, including daily dose, TBiL, BUN, and Scr.

#### Serum Lipid Profiling Using LC-Q-TOF/MS

**Figure 1:**
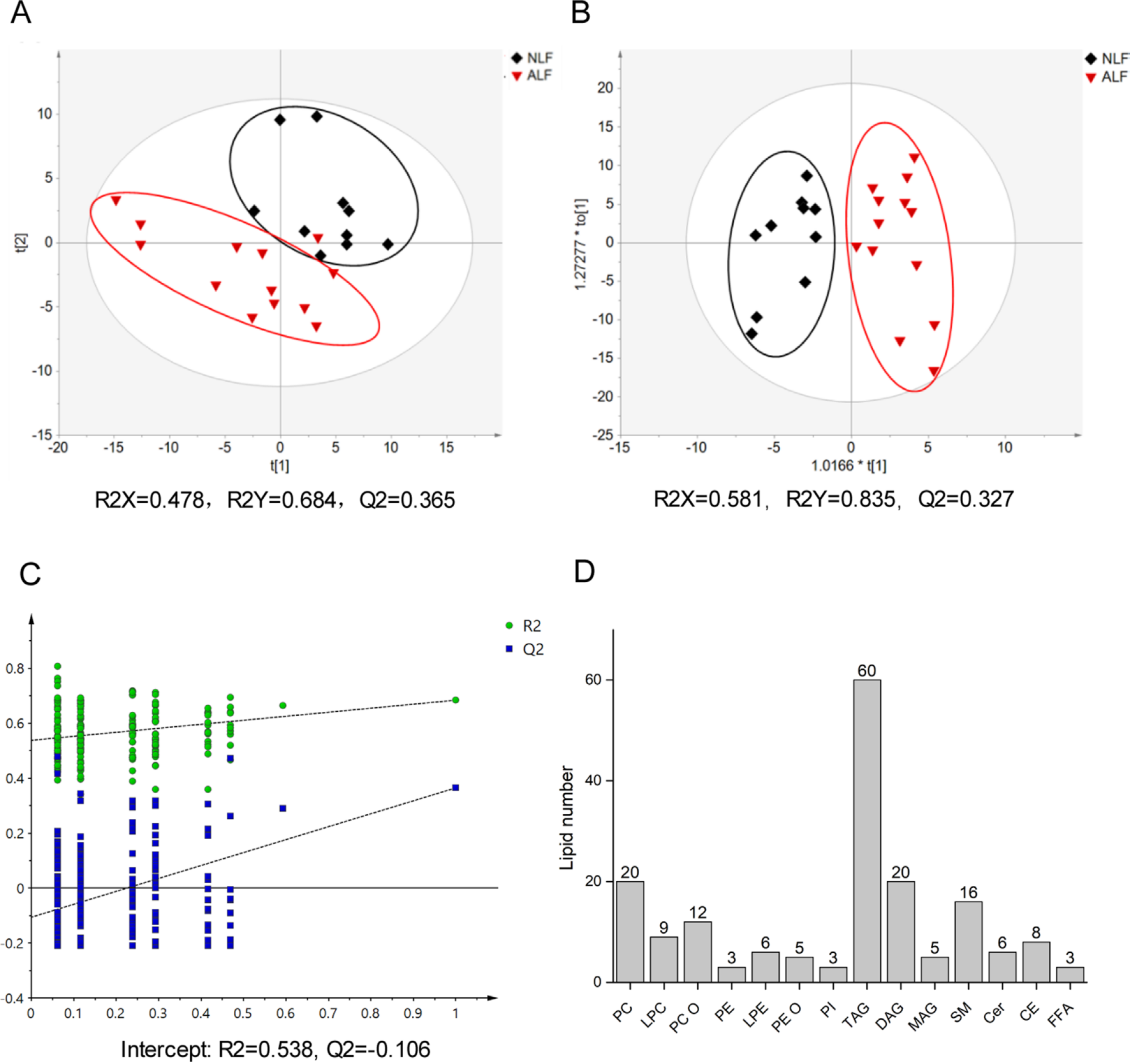
Score plot of the PLS-DA model **(A)** and the OPLS-DA model **(B)** based on UV scaling for ALF subject (*red dots*) and NLF (*black dots*) separation. **(C)** Cross-validation plot of PLS-DA mode with 200 times permutation tests. **(D)** Number of detected lipids based on a nontargeted lipidomics strategy in patient serum.

To survey overall lipid changes, a nontargeted lipidomics method based on LC-Q-TOF/MS technology in both positive and negative ion modes was applied to discover serum lipid changes upon VPA-induced hepatotoxicity. Retention times and peak areas of all detected lipids in QCs were evaluated by their relative standard deviations (RSDs), and only those with an RSD below 30% were subjected to further analysis. According to exact mass, retention time, and MS/MS pattern, 176 lipids comprising 14 different lipid subclasses were identified with the established lipidomics analytical platform, with TAG and phosphatidylcholines (PC) as the most abundant classes (**Figure 1D**). Detailed information on *m/z*, retention time, and adduct ions of the identified lipids in serum is described in **Supplementary Table S2**.

To obtain a direct overview of systemic differences in serum lipid profile between ALF and NLF subjects, a PLS-DA model was used to investigate separation trends. As shown in **Figures 1A, C**, apparent separation illustrated a profound difference between ALF and NLF subjects at the lipid level. The PLS-DA model possessed two principal components in which R2X, R2Y, and Q2 were 0.478, 0.684, and 0.365, respectively. To improve group separation, an OPLS-DA model was established after UV scaling to provide an overview of lipid profiling between the ALF and NLF groups (**Figure 1B**).

Based on the relative intensities of the metabolites from the normalized spectrum, unpaired *t* test (*P* < 0.05) was used to reveal the significant differences in identified metabolites between VPA-induced ALF and NLF subjects, in combination with fold change > 1.5 and VIP value > 1.0. A total of 20 serum lipid metabolites were considered most responsible for observed differences between ALF and NLF subjects. Significant variables are summarized in **Table 2**, and lipidome data including formula, retention time, *m/z*, metabolite identification, and mass type were collected. To better understand the relationship between differential lipids and hepatotoxicity severity (ALT level), correlation analysis was performed between identified differential lipids and serum ALT (**Table 2**), demonstrating that a lipid metabolism disorder is closely correlated to the severity of hepatotoxicity with |*r*| > 0.42 (*P* < 0.05).

**Table 2:** Identification of differential lipids and correlation analysis in patient serum.

Differential lipids	Formula	RT (min)	Extract mass (Da)	Mass type	Correlation with ALT	*P*
LPC 16:0	C24H50O7NP	2.8	554.3453	[M+CH3COOH-H]^-^	−0.463	0.026
LPC 18:0	C26H54O7NP	3.8	582.3766	[M+CH3COOH-H]^-^	−0.475	0.022
LPC 18:1	C26H52O7NP	3.0	580.3609	[M+CH3COOH-H]^-^	−0.608	0.002
LPC 18:2	C26H50O7NP	2.5	578.3453	[M+CH3COOH-H]^-^	−0.583	0.004
LPC 20:0	C28H58O7NP	4.8	610.4079	[M+CH3COOH-H]^-^	−0.426	0.043
SM 34:1;3	C39H79O7N2P	7.5	719.5698	[M+H]^+^	−0.514	0.012
SM 34:2;3	C39H77O7N2P	6.8	717.5541	[M+H]^+^	−0.536	0.008
SM 36:2;2	C41H81O6N2P	8.2	729.5905	[M+H]^+^	−0.550	0.007
SM 38:2;2	C43H85O6N2P	9.0	757.6218	[M+H]^+^	−0.574	0.004
SM 40:2;2	C45H89O6N2P	9.8	785.6531	[M+H]^+^	−0.528	0.010
Cer 42:1;2	C42H83N1O3	10.9	650.6446	[M+H]^+^	−0.494	0.017
Cer 42:2;2	C42H81N1O3	10.6	648.6289	[M+H]^+^	−0.505	0.014
TAG 56:4	C59H106O6	13.4	928.8328	[M+NH4]^+^	+0.731	<0.001
TAG 56:5	C59H104O6	13.2	926.8171	[M+NH4]^+^	+0.642	0.001
TAG 56:6	C59H102O6	13.0	924.8015	[M+NH4]^+^	+0.579	0.004
TAG 58:5	C61H108O6	13.5	954.8484	[M+NH4]^+^	+0.711	<0.001
TAG 58:6	C61H106O6	13.3	952.8328	[M+NH4]^+^	+0.506	0.014
TAG 58:7	C61H104O6	13.1	950.8171	[M+NH4]^+^	+0.423	0.034
TAG 60:8	C63H106O6	13.2	976.8328	[M+NH4]^+^	+0.494	0.017
TAG 60:9	C63H104O6	13.0	974.8171	[M+NH4]^+^	+0.500	0.015

**Figure 2:**
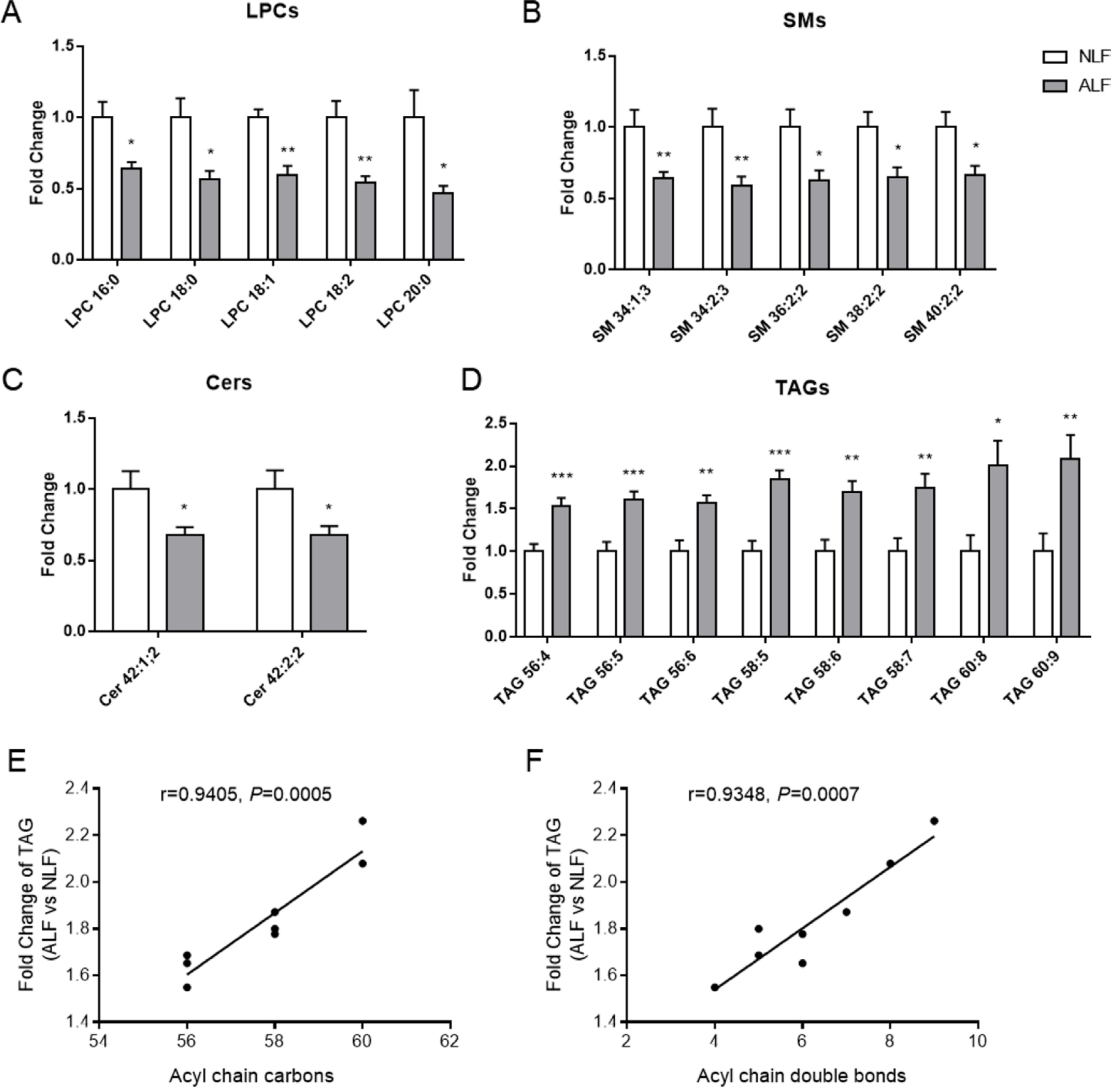
Relative levels of LPCs **(A)**, SMs **(B)**, Cers **(C)**, and TAGs **(D)** in patient serum. Relationship between fold change of TAG (ALF subjects vs controls) and lipid carbon number **(E)** or double bond content **(F)**. All data presented were normalized to the average of each control (adjusted average control value is 1). Values are presented as mean ± SD; *P < 0.05 and **P < 0.01 indicate statistical significance between NLF subjects (*n* = 10) and ALF subjects (*n* = 13).

From the identified lipid molecules, serum LPCs, SMs, and Cers were reduced, whereas TAGs were increased in ALF subjects as compared to NLFs (**Table 2** and **Figures 2A–D**). Thus, these lipid species are considered potential novel plasma biomarkers for hepatotoxicity induced by VPA; we therefore subjected them to further statistical analysis. As shown in **Figures 2A–D**), significantly lower levels of the selected LPCs (16:0, 18:0, 18:1, 18:2, and 20:0) were observed in VPA-induced ALF. Potential alterations by VPA-induced hepatotoxicity in signaling lipids were also found: SM levels (34:1;3, 34:2;3, 36:2;2, 38:2;2, and 40:2;2) with an even summed carbon number were in ALF subjects, and Cer levels (42:1;2 and 42:2;2) were much lower in ALF subjects than those in NLFs. Of the 60 TAG species identified in positive mode as [M+NH4]^+^ ions, we found 14 TAGs that were significantly increased in ALF subjects as compared to NLFs (fold change > 1.5, *P* < 0.05; data not shown). Moreover, an increasing trend of eight TAGs with an even summed carbon number over 56 was observed in VPA-induced ALF (fold change > 1.5, *P* < 0.05, VIP > 1), and these TAGs showed a pronounced positive relationship with ALT value (**Table 2** and **Figure 2D**). In addition, a positive relationship between fold change, and carbon number and double bond content was also present among TAGs (**Figures 2E, F**). Besides, ROC curve was used to identify the specificity and sensitivity of potential biomarkers in diagnosis of VPA hepatotoxicity (**Supplementary Figure S1**). All of the area under curves (AUCs) of candidate biomarkers exceeded 0.750, which indicated a good prediction of hepatoxicity. These results showed that VPA-induced hepatotoxicity can change signal and energy lipid levels and that different lipids could be candidate biomarkers to predict the liver injury in epileptic patients administered with VPA.

### Lipidomics Analysis in VPA-Induced Hepatotoxicity Cells

#### VPA-Induced L02 Cell Damage

**Figure 3:**
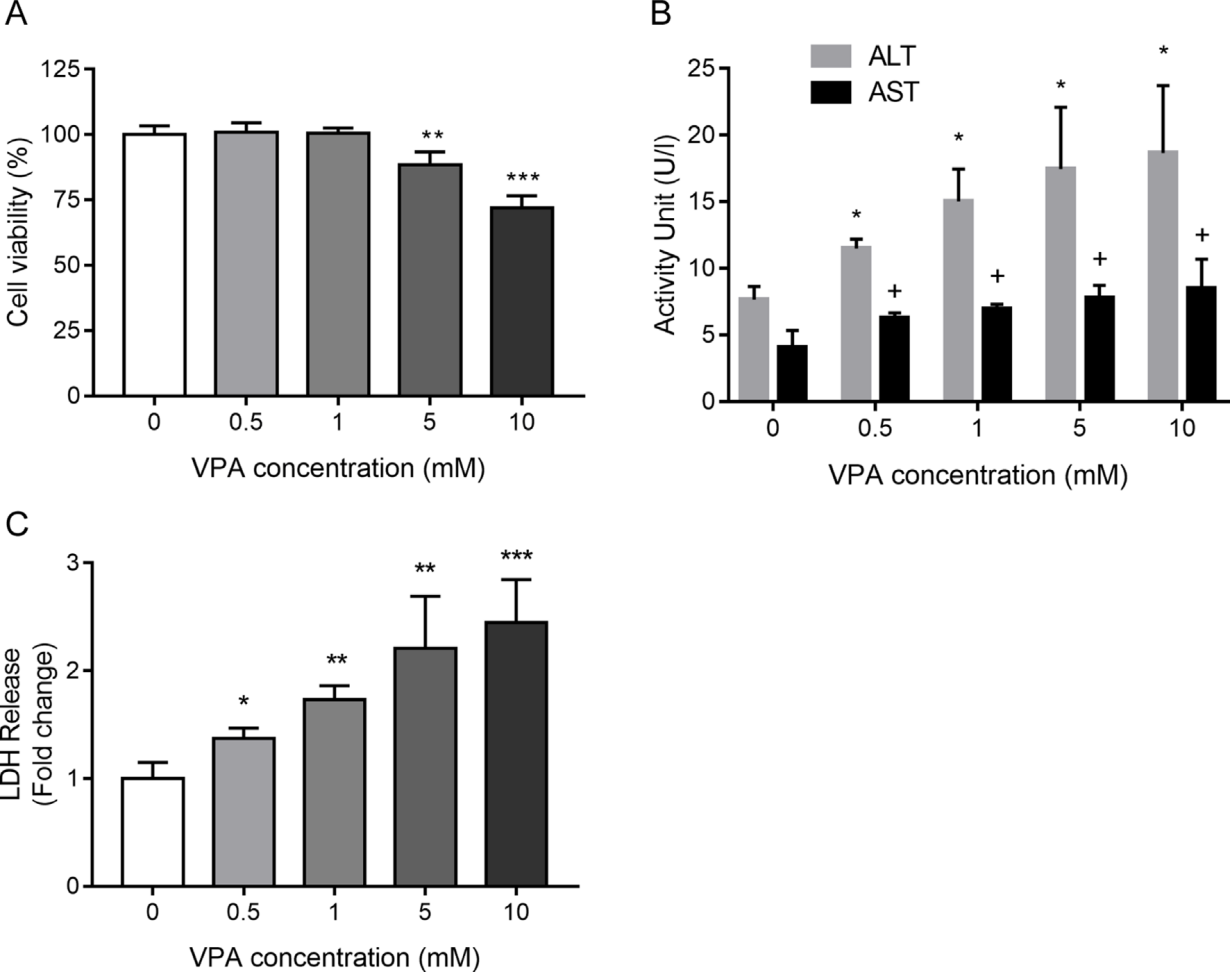
The toxic effect of VPA on cell viability **(A)**, ALT and AST activity **(B)**, and the activity of LDH **(C)** in the culture medium collected from L02 cells treated by VPA. L02 cells were exposed to different concentrations of VPA (0, 0.5, 1, 5, and 10 mM) for 24 h *in vitro*. Data are expressed as mean ± SD (*n* = 3); *P < 0.05, **P < 0.01, and ***P < 0.001 compared with control group regarding cell viability and ALT levels; ^#^ *P* <0.05 compared with control group regarding AST levels.

To examine cellular toxicity effects induced by VPA, an MTT assay was performed with different concentrations of VPA applied to L02 cells for 24 h. As shown in **Figure 3A**, VPA at concentrations greater than 5 mM significantly inhibited cell viability relative to control cells (*P* < 0.05). ALT and AST activities were significantly elevated in the VPA treatment group in a dose-dependent manner as compared to the control group (**Figure 3B**). The measurement of the leakage of LDH in the culture medium was used to evaluate plasma membrane integrity. The measurement of the leakage of LDH in the culture medium was used to evaluate plasma membrane integrity. The activity of LDH further increased with the increased concentration of VPA (**Figure 3C**), which confirms hepatotoxicity induced by VPA.

#### Lipidomics Analysis of VPA-Induced Cytotoxicity in L02 Cells

**Figure 4:**
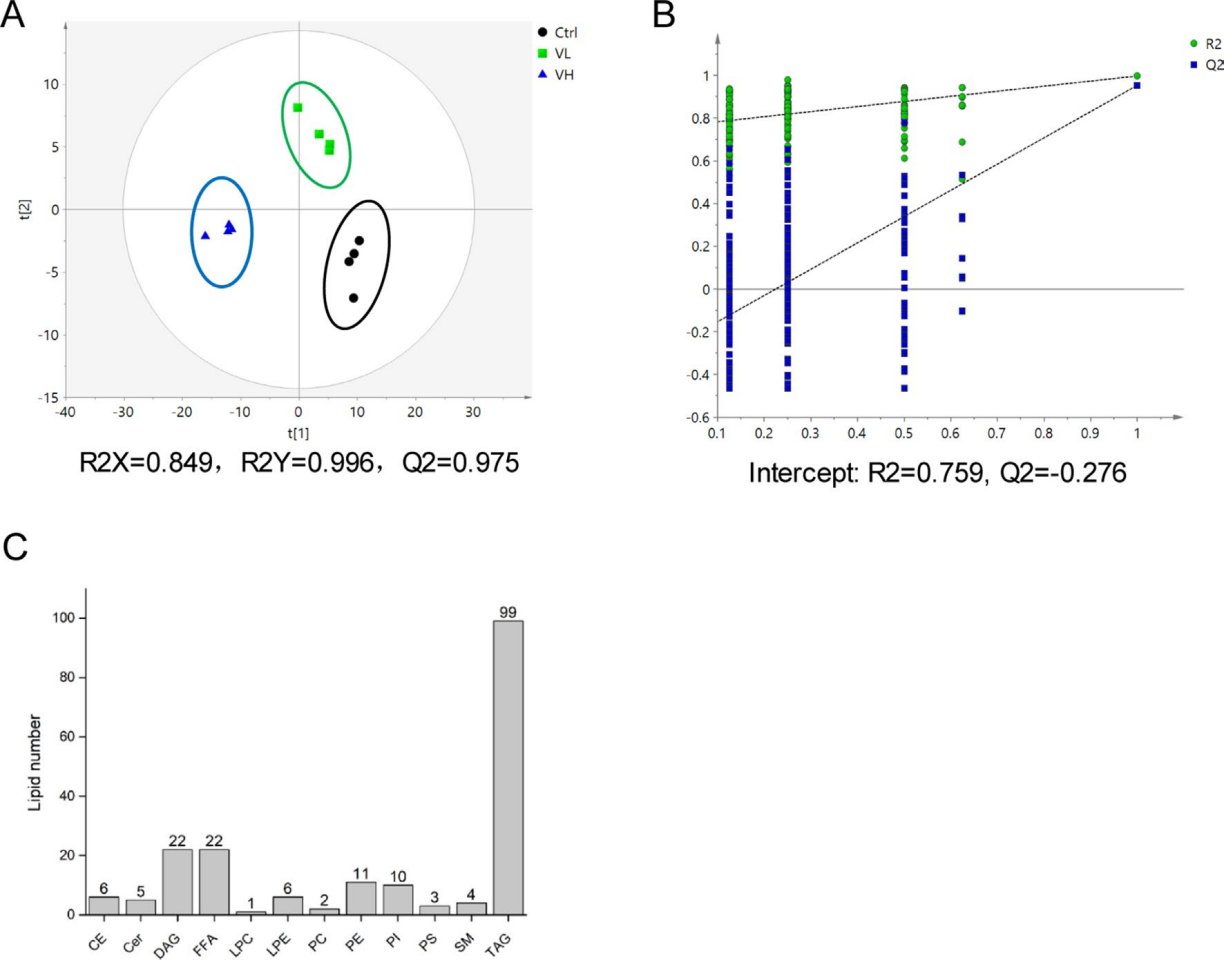
**(A)** Score plot of the PLS-DA model based on UV scaling for the VL group (*green boxes*), VH group (*blue triangles*), and control (*black dots*) separation (*n* = 4). **(B)** Cross-validation plot of PLS-DA mode with 200 times permutation tests. **(C)** Number of detected lipids based on a nontargeted lipidomic strategy in L02 cells treated with VPA.

Using lipidomic profiling of VPA-treated L02 cells, we found the presence of 191 lipids comprising 12 different lipid subclasses (**Figure 4C**). TAG, DAG, and FFA were the most abundant classes *via* cellular lipidomic profiling. Then, supervised PLS-DA models were established to maximize differences in metabolic profile between control and VPA groups as well as to facilitate screening for lipid marker metabolites (**Figures 4A, B**), in which R2X = 0.849, R2Y = 0.996, and Q2 = 0.975. Control and VPA-treated cells were clearly separated based on the score plot, indicating that these groups were in distinct lipidomic profiles.

**Figure 5:**
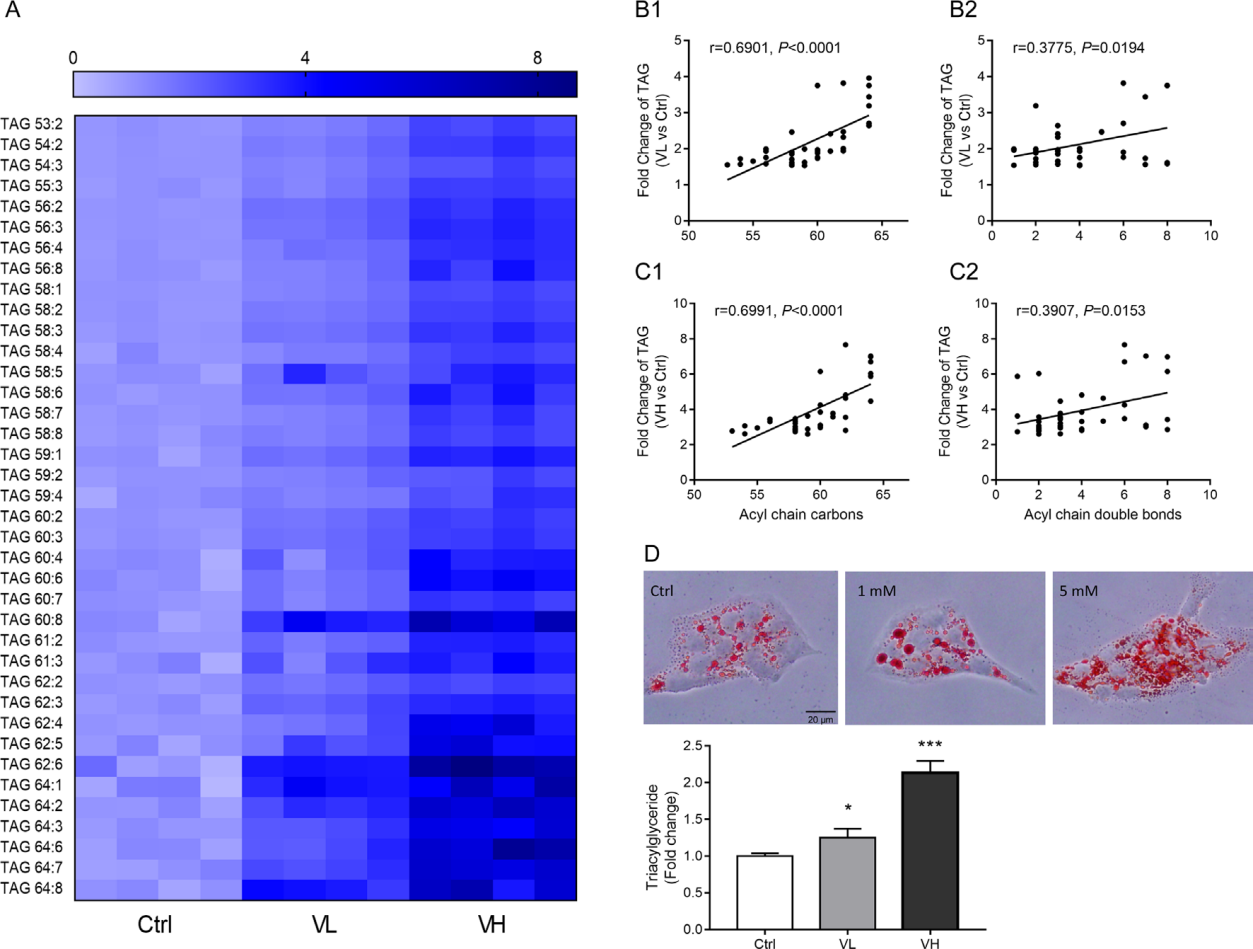
Long-chain TAG significant elevated in VPA-treated hepatic L02 cells for 24 h. **(A)** Heatmap of L02 cells lipids showing significant differences in both VL (VPA 1 mM) and VH (VPA 5 mM) groups as compared to the control group (*P* < 0.05, VIP > 1, fold change >1.5). Correlation between TAG and carbon number fold change (**B1** for the VL group; **C1** for the VH group) and double bond content (**B2** for the VL group; **C2** for the VH group). Correlation factor (*r*) and *P* value were estimated with Pearson’s correlation analysis. **(D)** Oil Red O staining and TAG assays revealed that VPA induced hepatic steatosis in L02 cells. Staining images are displayed at 400× magnification. Values are presented as mean ± SD (*n* = 4); *P < 0.05, ***P < 0.001 compared with the control group.

Next, differential lipids were investigated between VL/VH groups and the control group using *t* tests (*P* < 0.05; FDR significance criterion = 0.05) and VIP > 1.0 (PLS-DA) in combination with fold change > 1.5. The largest differences in both magnitude and significance of the associations (as reflected by fold change and *P* value, respectively) were noted among TAGs. Forty-eight and 87 lipids showed significant differences in the VL group and VH group, respectively, as compared to control cells. Thirty-eight lipids were found to be in common between the two comparisons; detailed information on these species is shown in a heatmap (**Figure 5A**). It can be clearly seen that most TAGs with a summed carbon number over 53 are markedly increased in VPA groups. After VPA treatment, we found a positive relationship in which TAGs with relatively higher carbon numbers and double bond content are most significantly elevated in VPA-treated cells (VL and VH groups) compared with controls (**Figures 5B, C**), which is consistent with patient serum results (**Figures 2E, F**). Similarly, treatment with VPA significantly increased hepatic intracellular lipid content, which was confirmed by Oil Red O (red) staining as shown in **Figure 5D**, and this effect was especially significant in the VH group. Treatment with VPA 1 mM and 5 mM for 24 h increased the intracellular lipid content by about 25% and 100%, respectively, in comparison with controls.

No significant differences in total FFAs between VPA and control groups were found *via* PCA analysis. When hepatic cell FFA levels were grouped according to FFA saturation, an interesting pattern emerged (**Supplementary Figure S2**): cell saturated FA (SFA) levels were not significantly altered by VPA treatment (**Supplementary Figure S2B**); however, a slightly increased trend was found in monounsaturated FA (MUFA) and polyunsaturated FA (PUFA; **Supplementary Figures S2C, D**). We also found that the more long-chain FFA (FFA20-26) accumulated in VPA groups, but not FFA with 14–18 carbons (FFA14–18) (**Supplementary Figure S2E**).

### VPA-Induced Dysregulation of Lipid Metabolism Genes

**Figure 6:**
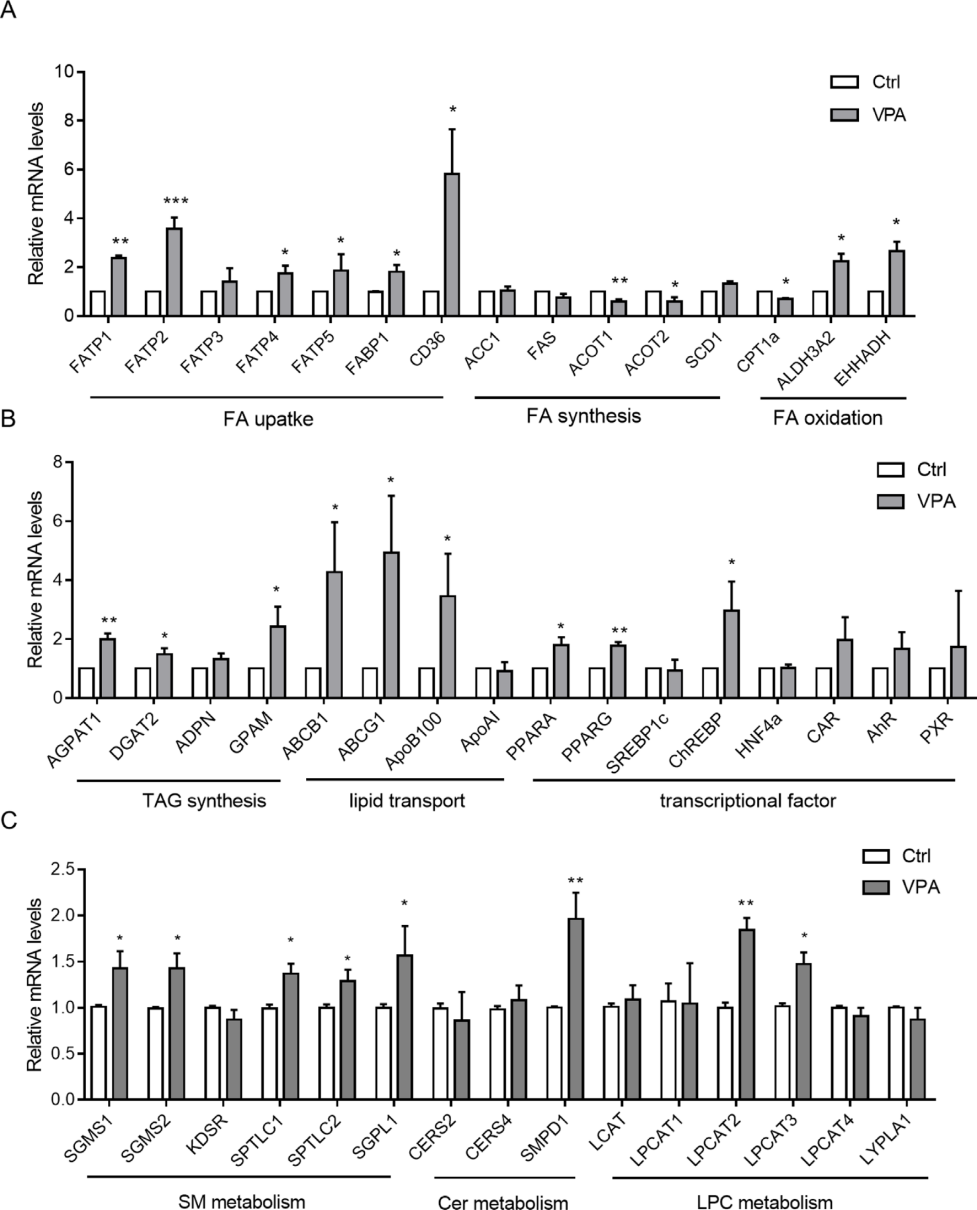
Hepatic mRNA levels of genes associated with metabolism of FA **(A)**; TAG, transporters, and transcriptional factors **(B)**; and SM, Cer, and LPC **(C)** in L02 cells treated with VPA. Target gene mRNA levels are normalized to those of GAPDH and subsequently normalized to those of control groups. Values are presented as mean ± SD (*n* = 3); *P < 0.05, **P < 0.01, ***P < 0.001 compared with the control group.

According to cytotoxicity results and the VPA concentration in the clinic, the VPA concentration (1 mM) was used to investigate the effect of VPA on lipid metabolism. Hepatic gene expression profiles were determined by qPCR for control and VPA-treated (1 mM) L02 cells. To better understand the observed changes in lipid metabolites, key enzymes and transcriptional regulators involved in hepatic lipid metabolism were investigated (**Figure 6**). Gene analysis included genes involved in FA uptake (*CD36*, *FATPs*, and *FABP1*), *de novo* lipogenesis (*ACC1*, *FAS*, *ACOT1*, *ACOT2*, and *SCD1*), FA beta-oxidation (*CPT1a*, *EHHADH*, and *ALDH3A2*), triglyceride synthesis and hydrolysis (*AGPAT1*, *DGAT2*, *ADPN*, and *GPAM*), lipid transport (*ABCB1*, *ABCG1*, *ApoB100*, and *ApoAI*), transcriptional regulation of lipid metabolism (*PPARA*, *PPARG*, *SREBP1c*, *ChREBP*, *HNF4a*, *CAR*, *AhR*, and *PXR*), LPC metabolism (*LCAT*, *LPCAT1-4*, and *LYPLA1*), SM metabolism (*SGMS1*, *SGMS2*, *SPTLC1*, *SPTLC2*, *KDSR*, and *SGPL1*), and ceramide synthesis (*CERS2*, *CERS4*, and *SMPD1*).

With regard to FA uptake genes, we observed significantly increased *CD36*, *FATP*s, and *FABP1* expression in the VPA-treated group (**Figure 6A**), all of which play critical roles in FA recognition and fat perception (Pepino et al., 2012). Expression of *EHHADH* and *ALDH3A2* (PPARA targets), regarded important genes for FA oxidation (Houten et al., 2012), was upregulated in the VPA-treated group, and that of *CPT1*α was downregulated. We also found that the expression of triglyceride synthesis and hydrolysis genes (*AGPAT1*, *DGAT2*, and *GPAM*) was significantly increased in VPA-treated cells. Lipid transport genes (*ABCB1*, *ABCG1*, and *ApoB100*) were also upregulated by VPA treatment, as *de novo* lipogenesis genes (*ACOT1* and *ACOT2*) were downregulated, while *SCD1* was upregulated.

Several transcriptional regulators associated with lipid metabolism in liver (*PPARA*, *PPARG*, etc.) were also tested (**Figure 6B**). PPARA signaling is typically associated with increased FA oxidation (Kersten and Stienstra, 2017), and the gene was upregulated in the VPA treatment group. *PPARG*, which promotes lipogenesis, was significantly upregulated, as well as *ChREBP*. Genes in the *de novo* lipogenesis pathway are regulated in part by *SREBP1c*, but we observed no change in the expression of this gene or in genes it regulates (*ACC1* and *FAS*).

Levels of mRNAs encoding lysophosphatidylcholine acyltransferases (*LPCAT*) 1 to 4 and lysophospholipase A1 (*LYPLA1*), which are involved in LPC metabolism (Farooqui et al., 1984; Aoki, 2004; Shindou and Shimizu, 2009), were determined in liver cells. Hepatic *LPCAT2* and *LPCAT3*, which encode key enzymes that convert LPC into PC, were increased by about 1.8-fold and 1.5-fold, respectively, in VPA-treated cells (**Figure 6C**). Proinflammatory cytokines, such as TNFα, IL6, and TGFβ, are among the major contributors to liver injury pathogenesis (Day, 2006; Neuschwander-Tetri, 2010; Tilg and Moschen, 2010). We found that hepatic *TNF*α, *IL6*, and *TGF*β mRNA levels were increased in VPA-treated L02 cells (**Supplementary Figure S3**). A prior study showed that these cytokines significantly induce *LPCAT* mRNA expression (Tanaka et al., 2012). Thus, hepatic upregulation of proinflammatory cytokines and the accompanying induction of *LPCAT2/3* are considered among the maim causes of serum LPC reductions (Tanaka et al., 2012).

Sphingolipid and ceramide metabolism mechanisms are complex, and SM is mainly regulated by SM synthase (SGMS) and sphingomyelin phosphodiesterase (SMPD, also known as sphingomyelinase) (Hannun, 1994). *SGMS1*,* SGMS2*, *SPTLC1*,* SPTLC2*, and *SGPL1* mRNA levels were slightly increased (1.3-fold) after VPA exposure (**Figure 6C**). Acidic sphingomyelinase *SMPD1* mRNA levels were markedly elevated about onefold. The expression of other sphingolipid and ceramide metabolism genes (*KDSR*, *CERS2*, and *CERS4*) was not altered. These results suggest that hepatic disruption of SM-Cer homeostasis occurs after VPA exposure.

### The Akt–PPARγ Pathway Participates in VPA-Induced Lipid Accumulation

**Figure 7:**
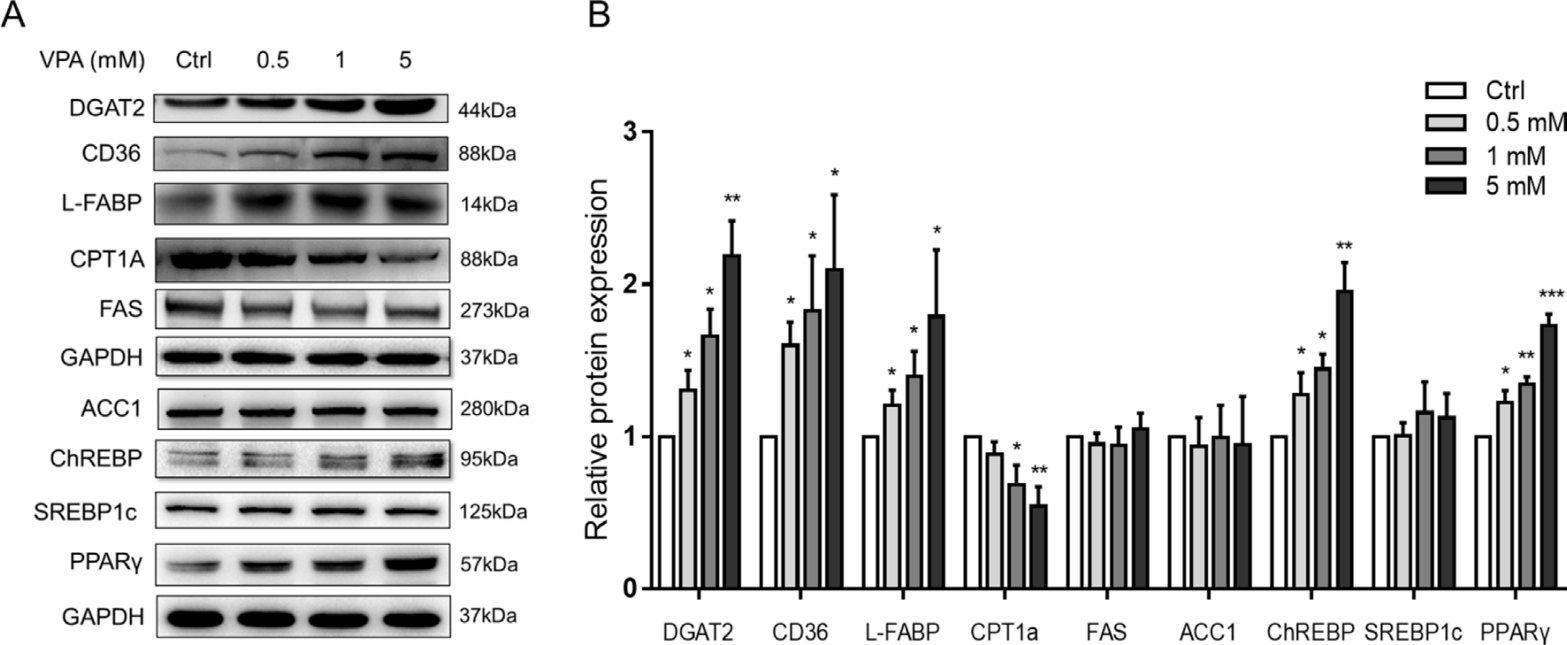
Protein expressions of lipid metabolism genes following treatment with VPA. **(A)** L02 cells were treated for 24 h with VPA (0, 0.5, 1, and 5 mM). Whole-cell extracts were harvested and the expression levels of indicated genes and the internal control (GAPDH) were analyzed by Western blot. A representative blot is shown. **(B)** Band densities were quantified using ImageJ Software; band density of control groups was set at 1.00. Values are presented as mean ± SD (*n* = 3); *P < 0.05, **P < 0.01, ***P < 0.001 compared with the control group.

We also tested the lipid-metabolism-related protein expression in L02 cells (**Figure 7**). Western blot analysis of the VPA-treated L02 cells showed that protein expression of DGAT2, CD36, L-FABP, ChREBP, and PPARγ increased significantly in a dose-dependent manner. However, the fatty acid synthesis proteins, such as FAS, ACC1, and SREBP1c, showed no changes. The expression of CPT1A, a major enzyme for mitochondrial fatty acid β-oxidation (Aires et al., 2010), was decreased in VPA-treated groups. From the results, the FA uptake and TAG synthesis might contribute to the accumulation of TAG with higher carbon number. DGAT2 is a rate-limiting enzyme that esterifies diacylglycerol in the final step of the hepatic TAG biosynthetic pathway (Zammit, 2013). CD36 plays an important role in long-chain fatty acids uptake in liver and was associated with hepatic steatosis (Wilson et al., 2016). PPARγ is a member of the nuclear receptor superfamily of ligand-activated transcription factors, which is crucial for lipogenesis (Rogue et al., 2010). Fatty acid translocase CD36 could be regulated by the PPARγ pathway (Rogue et al., 2010), and several studies reported the co-expression of DGAT2 and PPARγ (Iqbal et al., 2016; Bai et al., 2017).

**Figure 8:**
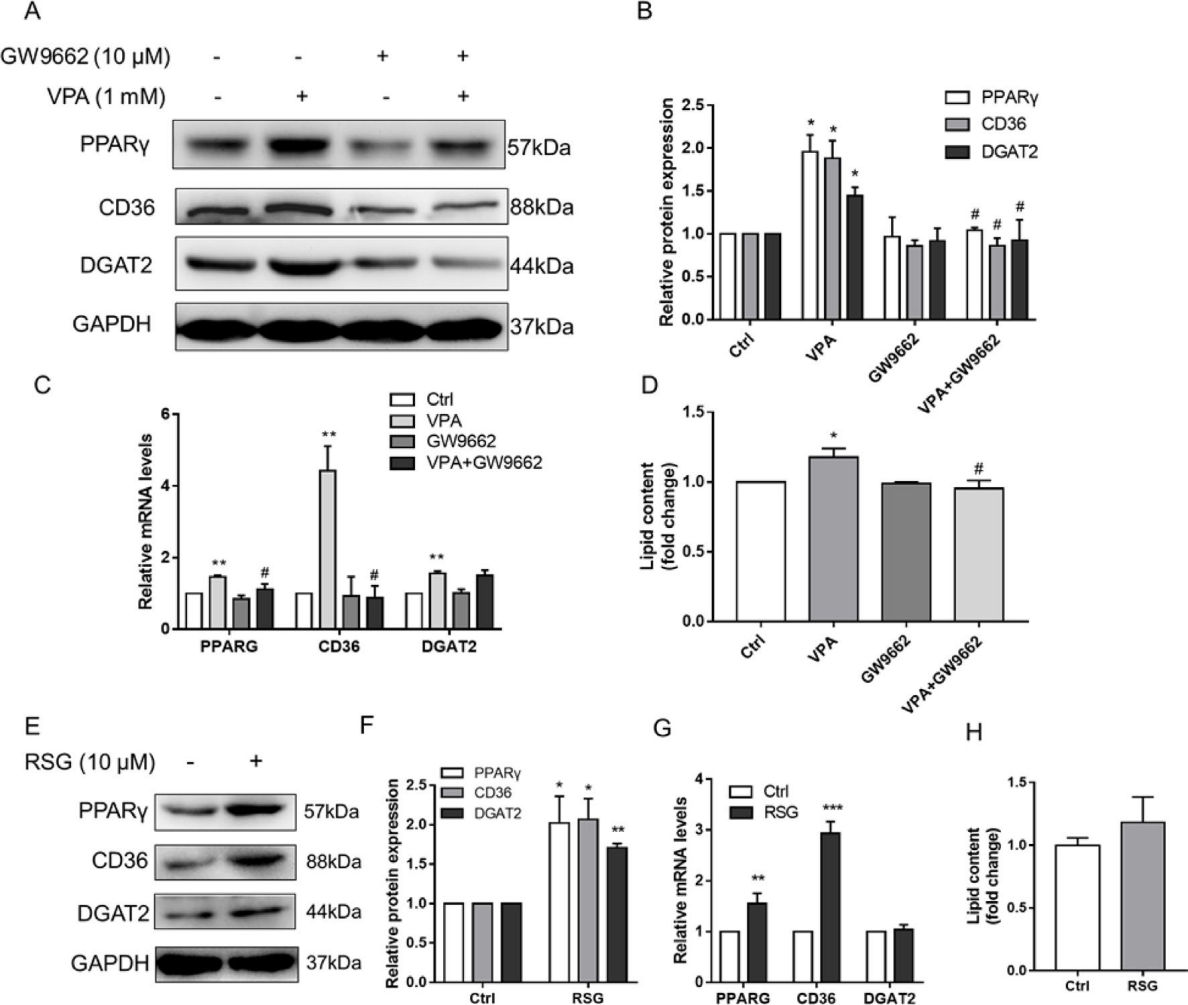
Effect of PPARγ pathway on VPA-induced lipid accumulation. L02 cells were pretreated with a PPARγ antagonist (GW9662, 10 µM) for 1 h and then treated with VPA (1 mM) for 24 h **(A–D)** or treated with PPARγ agonist (rosiglitazone, 10 µM) for 24 h **(E–H)**. The protein expression levels of PPARγ, CD36, DGAT2, and the internal control (GAPDH) were analyzed by Western blot **(A, B, E, F)**. A representative blot is shown. The mRNA levels of PPARG, CD36, and DGAT2 were analyzed by real-time PCR **(C, G)**. The lipid contents were determined by kit normalized by protein **(D, H)**. Results are expressed as mean ± SD (*n* = 3); *P < 0.05, **P < 0.01, ***P < 0.001 compared with the control group; ^#^ *P* < 0.05 compared with VPA group.

**Figure 9:**
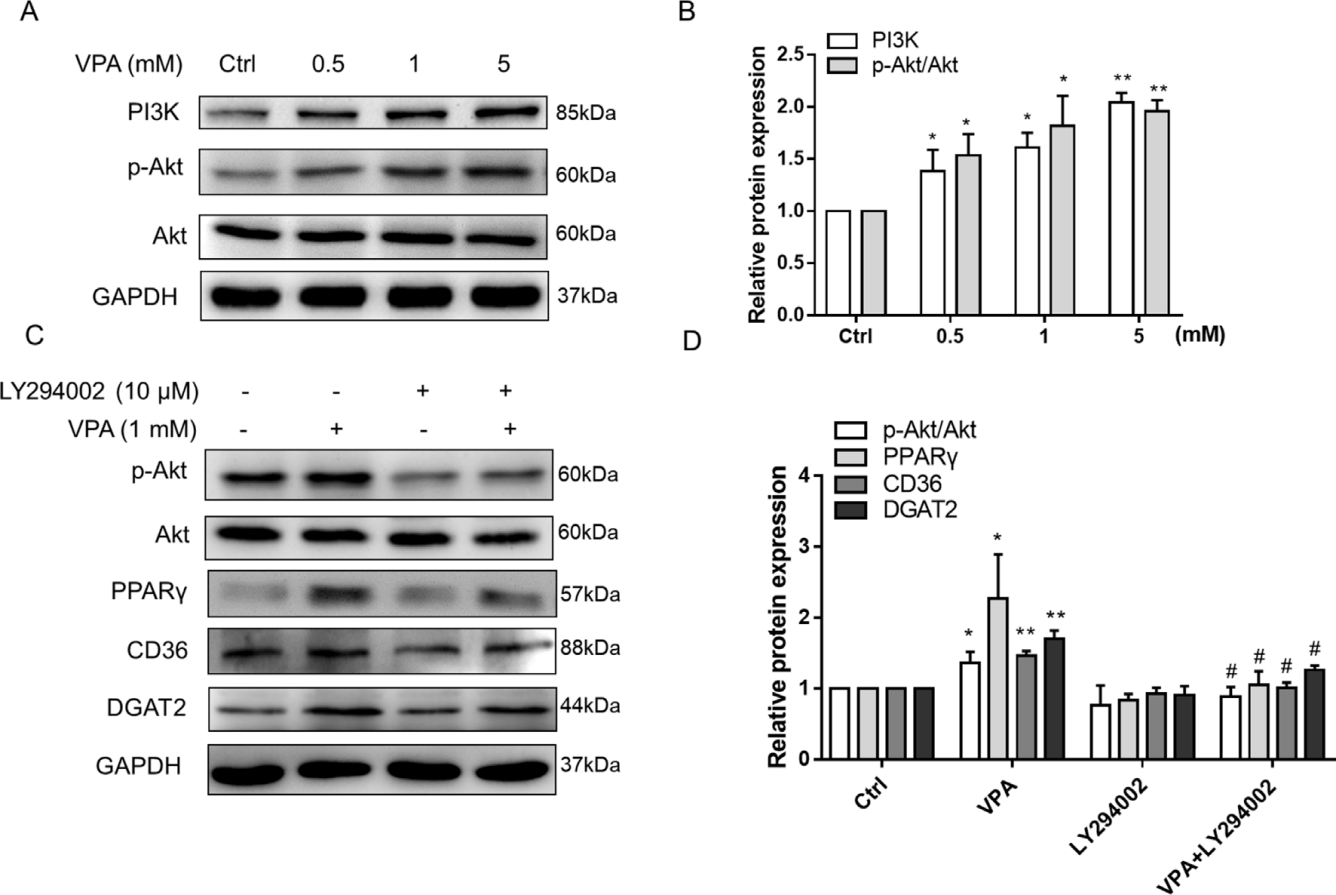
Effect of VPA-induced PI3K/Akt activation on PPARγ pathway. L02 cells were treated with VPA (0, 0.5, 1, and 5 mM) for 24 h, and the protein expression of PI3K, p-Akt, and Akt was determined. A representative Western blot is shown **(A, B)**. L02 cells were pretreated with a PI3K/Akt pathway inhibitor (LY294002, 10 µM) for 1 h and then treated with VPA (1 mM) for 24 h **(C, D)**. Then, the protein expressions of indicated genes were measured by Western blot experiments. Results are expressed as mean ± SD (*n* = 3); *P < 0.05, **P < 0.01 compared with the control group; ^#^ *P* < 0.05 compared with VPA group.

We hypothesized that PPARγ-mediated fatty acid uptake and TAG synthesis might be involved in VPA-induced hepatic steatosis. L02 cells were pretreated with selective PPARγ antagonist (GW9662, 10 μM) for 1 h and then exposed by VPA (1 mM) for 24 h. The Western blot results showed that GW9662 could obviously inhibit the expression of PPARγ, and the induction of CD36 and DGAT2 protein expression by VPA was abolished (**Figures 8A, B**). Besides, the mRNA level of PPARG and CD36 was reversed by GW9662, except DGAT2 (**Figure 8C**), and lipid accumulation induced by VPA was almost totally abrogated by GW9662 (**Figure 8D**). To further determine whether upregulation of CD36 and DGAT2 is mediated by the PPARγ pathway, L02 cells were treated by a selective PPARγ agonist (rosiglitazone, 10 μM) for 24 h. Although no significant lipid accumulation was found after rosiglitazone treatment in L02 cells, Western blot results showed that rosiglitazone significantly increased the protein expression of PPARγ, CD36, and DGAT2 (**Figures 9E, F**). The mRNA level of PPARG and CD36 was upregulated by rosiglitazone (**Figure 9G**). However, rosiglitazone did not change DGAT2 mRNA level, suggesting the involvement of posttranscriptional regulation. These evidences revealed that VPA induced DGAT2 and CD36 protein upregulation through PPARγ pathway.

Akt pathway acts as a critical signaling regulator triggering a number of insulin-stimulated effects and contributes to the lipid deposition in liver (Wang et al., 2017; Liao et al., 2018). A previous study has reported that VPA has the ability to upregulate the expression of p-Akt in U87 cells (Zhang et al., 2016), which was also observed in our results (**Figures 9A, B**). We also found that VPA could increase the mRNA level of GLUT4 (**Supplementary Figure S4**), which is the insulin-regulated glucose uptake transporter (Beg et al., 2017). Thus, Akt pathway might participate in the lipid accumulation induced by VPA. To investigate the effect of Akt pathway on VPA-induced lipid accumulation, Akt pathway selective inhibitor LY294002 was used, and Western blots presented that LY294002 obviously decreased the phosphorylation of Akt in the presence of VPA, and the protein expression of PPARγ, CD36, and DGAT2 induced by VPA was significantly abrogated (**Figures 9C, D**). These results indicate that VPA could upregulate PPARγ pathway through Akt signaling pathway.

## Discussion

VPA-induced hepatotoxicity is one of the most frequent and severe ADRs that limit the continual use of VPA, with much higher incidence in children (Dreifuss et al., 1987; Dreifuss et al., 1989; Nanau and Neuman, 2013). Furthermore, a greater risk of developing NAFLD has been found in patients treated with VPA (Luef et al., 2009). Thus, it is necessary to gain an understanding of dysregulated lipid metabolism in mechanism of VPA-induced hepatotoxicity. In this study, we performed lipidomics analysis of serum collected from pediatric epileptic patients with VPA hepatotoxicity and hepatic L02 cells treated by VPA. Although hepatic L02 cells with CYP450 enzyme activities, only about 10% of VPA was metabolized by CYP450-mediated oxidation (Ghodke-Puranik et al., 2013). Besides, studies reported that the metabolites of VPA levels in liver were much lower than those induced lipid accumulation in rat hepatocytes (Tong et al., 2005; Surendradoss et al., 2012). Thus, little effect of metabolites might influence the lipid metabolism disrupted by VPA in hepatic cells.

To our knowledge, this is the first study to characterize detailed changes using a large panel of lipid molecular species from different lipid subclasses upon VPA hepatotoxicity. The interpretation of these studies was extended by performing a systematic analysis of gene expression in VPA-treated hepatic L02 cells, targeting genes involved in the lipid pathways studied. This unified approach allows us to interpret changes in lipid levels and gene expression together, providing a mechanistic understanding of the development of VPA-induced hepatotoxicity.

### Increased TAG Levels and Disrupted Lipogenic Gene Expression

It has been proven that VPA treatment confers a higher risk of hyperlipidemia in epileptic patients (Chang et al., 2009; Chang et al., 2010). Elevated plasma lipid levels, such as TAGs, are inversely associated with the resolution of non-alcoholic steatohepatitis (Corey et al., 2015a; Corey et al., 2015b). Moreover, previous studies have shown that VPA therapy appears to be associated with an increased risk of developing NAFLD (Luef et al., 2009; Verrotti et al., 2009; Farinelli, 2015).

The present lipidomics analysis revealed reduced LPC, SM, and Cer lipid levels in ALF subjects, whereas TAGs increased substantially. TAG, as the most abundant lipid species, represents a major source of energy and constitutes a critical component of lipoproteins (Nordestgaard and Varbo, 2014). In our study, a total of 60 TAGs were identified in serum samples, and 8 TAGs showed significant increases in ALF subjects (fold change > 1.5, *P* < 0.05) and pronounced positive relationships with ALT levels (**Table 2**). As for lipidomics in hepatic L02 cells, TAGs with a summed carbon number over 53 also presented significant increases in the VPA group (**Figure 5A**). Additionally, TAGs with higher carbon numbers and double bond content showed higher fold changes in both serum (**Figures 2E, F**) and L02 cells (**Figures 5B, C**). These TAGs therefore might be associated with an increased risk of VPA-induced hepatotoxicity. Notably, it has been demonstrated that a pronouncedly elevated TAG level is a risk factor for cardiovascular disease, as a side effect of VPA treatment (Verrotti et al., 2010; Fang et al., 2012).

These findings can be understood in the context of our gene expression data in lipid metabolism. VPA could enhance the expression of FA transporter genes *CD36*, *FATPs*, and *FABP1* (PPARG targets), which contribute to FFA uptake from the circulation by liver (Ge et al., 2010; Bai et al., 2017). Significantly increased expression of triglyceride synthesis genes (*AGPAT1*, *DGAT2*, *GPAM*, and *SCD1*) were also observed in VPA group cells, which are known to be directly involved in hepatic triglyceride synthesis (Anderson and Borlak, 2008; Shindou and Shimizu, 2009). The lipid transporters (*ABCB1*, *ABCG1*, and *ApoB100*), regarded as lipid efflux transports that export lipids from the liver into circulation (Borst and Elferink, 2002; Engel et al., 2006), showed increased expression in the VPA group. Finally, FA oxidation genes like *EHHADH* and *ALDH3A2* (PPARA targets) were upregulated in the VPA treatment group, which might promote feedback of high hepatic FA uptake. However, the action of CPT1A is the primary regulated step in the mitochondrial oxidation of long-chain fatty acids in hepatocytes under most physiological circumstances (Drynan et al., 1996), as CPT1A was significant inhibited by VPA (Aires et al., 2010). Regarding transcription factor genes regulating lipid metabolism, increased expression of *PPARG* in the VPA group is associated with the excessive hepatic triglyceride accumulation (Berger and Moller, 2002) and mediates a shift from fatty acid oxidation to *de novo* lipid synthesis (Anderson and Borlak, 2008). PPARA is a sensor of PUFAs and regulates fatty acid oxidation (Sonoda et al., 2008). Previous studies reported that the inhibition of mitochondrial β-oxidation and upregulation of FA transporter by VPA lead to fatty acid accumulation in cells (Silva et al., 2008; Bai et al., 2017). Due to the toxic environment produced by accumulated lipids, upregulation of PPARA target genes could be interpreted as an adaptive response to increased FA uptake and lipid synthesis (Hall et al., 2010; Rakhshandehroo et al., 2010; Vitins et al., 2014). These results suggest that VPA exposure markedly alters hepatic lipid homeostasis, leading to disrupted TAGs metabolism in both liver cells and circulation.

### Akt–PPARγ Pathway Mediated VPA-Induced Lipid Accumulation

PPARγ acts as a critical transcription factor in the regulation of lipid storage and of genes involved in energy storage and utilization (Rogue et al., 2010). As a target gene of PPARγ, hepatic CD36 is associated with human and murine NAFLD by enhancing the fatty acid uptake capacity (Wheeler and Gekakis, 2014; Wilson et al., 2016). DGAT2 is a rate-limiting enzyme that esterifies diacylglycerol in the final step of the hepatic TAG biosynthetic pathway (Zammit, 2013), and contributed to chronic alcohol-induced alcoholic fatty liver disease (Wang et al., 2010). VPA could induce the expression of PPARγ, CD36, and DGAT2 (**Figure 7**). Fatty acid uptake and TAG synthesis were involved in VPA-induced hepatic steatosis by increasing the expression of CD36 and DGAT2 (Bai et al., 2017). In this study, the protein expressions of CD36 and DGAT2 were proved to be regulated by PPARγ pathway, which indicated that PPARγ-mediated fatty acid uptake and TAG synthesis participated in VPA-induced lipid accumulation.

PI3K/Akt pathway also plays an important role in insulin-stimulated effects, including glucose uptake, glycogen synthesis, and cell differentiation, as well as lipid metabolism (Wang et al., 2017; Liao et al., 2018). Besides, it was reported that the stimulatory effect of insulin on lipid deposition is mediated by PI3K/Akt regulation of lipogenesis pathway, including PPARγ and its target genes (Han et al., 2015). Moreover, the overexpression of PPARγ in Akt-deficient mouse embryonic fibroblasts rescued their severe adipogenesis defect, which indicates the essential role of PPARγ induction downstream of Akt pathway (Peng et al., 2003). Other studies have reported that increased or inhibited mTOR activity, a downstream target of PI3K/Akt, impairs lipid homeostasis by regulating the lipogenic gene PPARγ (Kang et al., 2018; Ko et al., 2018). Glycogen synthase kinase-3β (GSK-3β) was one of the first described physiological targets of Akt and also participated in the regulation of PPARγ (Song et al., 2017; Wang et al., 2019). Our results also showed that VPA activated Akt pathway and increased the mRNA level of GLUT4, which performed glucose uptake from circulation (Beg et al., 2017), and suggested that activation of PPARγ pathway by VPA could be regulated by Akt pathway.

### Reduced Serum LPC Levels in Serum and Altered Gene Expression

LPCs are regarded as important modulators of various physiological functions including inflammation, cell proliferation, and tumor invasiveness (Oestvang and Johansen, 2006; Best et al., 2013). In this study, significantly lower LPC levels (LPC 16:0, 18:0, 18:1, 18:2, and 20:0) were found in ALF patients as compared to NLFs (**Figure 2** **A** and **Table 2**). These results are consistent with those of a previous study, in which VPA induced ALF using a metabolomic approach (Huo et al., 2014). Furthermore, several studies have reported that lower LPC levels were associated with hepatic cirrhosis, hepatocellular carcinoma, and cholestasis (Matsubara et al., 2011; Patterson et al., 2011). Tanaka et al. (2012) found that lower LPC levels were related to proinflammatory cytokines and bile acids in nonalcoholic steatohepatitis, which is a progressive form of NAFLD. The proinflammatory cytokines are upregulated in VPA-treated L02 cells (**Supplementary Figure S3**). Moreover, reduced serum LPC levels were also found in acetaminophen-, D-galactosamine-, and carbon tetrachloride (CCl_4_)-induced liver injuries (Cheng et al., 2009; Ma et al., 2014; Ishikawa et al., 2016). Although LPC itself has been reported to possess lipotoxic properties (Han et al., 2007; Kakisaka et al., 2012), reductions in serum LPC levels are likely the result of hepatic inflammation (Tanaka et al., 2012). Additionally, reductions in serum LPC concentrations are significantly correlated to hepatic upregulation of LPCAT (Tanaka et al., 2012), which is consistent with the increased *LPCAT2* and *LPCAT3* mRNA levels observed in VPA-treated L02 cells.

### Disrupted SM-Cer Homeostasis and Hepatic Gene Expression

Sphingolipids, including sphingomyelin and ceramide, play crucial roles as structural components of the membrane and in cellular signaling promoting events, such as cell division, differentiation, gene expression, and apoptosis (Marí and Fernández-Checa, 2007). It has been demonstrated that ceramides could inhibit insulin signaling and induce oxidation stress and inflammation, which participated in NAFLD development (Marí and Fernández-Checa, 2007; Gill and Sattar, 2009), although the mechanistic link between hepatic steatosis and ceramide remains unclear. The alcohol fed significantly affected the lipid metabolism in mouse, and it was found that specific Cer levels reduced in plasma but elevated in the liver (Clugston et al., 2011). Moreover, a previous study has reported that VPA increased ceramide and phytosphingosine levels in yeast cells (Jadhav et al., 2016). Our serum lipidomics analysis revealed that VPA-induced hepatotoxicity was related to the reduced Cer and SM levels. Ceramide production is mediated by *de novo* synthesis *via* serine palmitoyltransferase (SPTLC) and ceramide synthase (CERS), or by the hydrolysis of membrane sphingomyelin by sphingomyelin phosphodiesterase (Holland and Summers, 2008). From gene expression analysis, hepatic *SGMS1*, *SGMS2*, *SPMD1*, *SPLTC1*, and *SPLTC2* expression levels, as the key enzymes regulating Cers and SMs, were markedly induced after VPA exposure. Increased activity of SMPD1, which uses sphingomyelin as a substrate for ceramide synthesis, might lead to the reduced serum SM levels (Matsubara et al., 2011). Although we did not find any differences in cellular Cers or SMs due to the limited species that were able to be detected in cells (**Figure 4A**), hepatic disruption of SM-Cer homeostasis was also observed *via* serum lipidomics and cell gene expression analysis.

### Highlights and Limitations

This was the first study on VPA-induced hepatotoxicity in pediatric patients and hepatic cells using lipidomic profiling coupled with an investigation on changes in lipid metabolism. Several studies reported that *in vivo* mouse model was used to investigate the drug-induced toxicity (Jin et al., 2014; Vitins et al., 2014). However, the lipid species and lipid metabolism genes are different between mouse and human (Lian et al., 2018). Besides, *in vitro* studies of human liver cells, such as L02 cells, had been used for the drug-toxicity studies (Shi et al., 2016; Lei et al., 2017), including metabolomics. Cell omics studies are also highly efficient approaches for identifying toxic biomarkers and helping understand toxicity mechanisms. As known, ALT and AST levels are considered good indicators of liver cell damage. However, all of the liver functions only indicated the happened liver injury, which lack sensitivity and mechanism for VPA hepatotoxicity. In this study, TAGs containing higher carbon numbers and other lipids showed a good specificity and sensitivity to predict hepatotoxicity using Pearson correlation analysis and ROC curve.

There are some limitations of this study that should be considered. Because VPA-induced hepatotoxicity incidence is very low in pediatric patients, a limited number of subjects with hepatotoxicity were able to be enrolled in this study. Further validation research should be performed to confirm the results and the mechanism of VPA-induced hepatotoxicity.

## Conclusion

This study provides a novel understanding of dysregulated lipid metabolism in VPA-induced hepatotoxicity using lipidomics profiling and found that TAGs containing higher carbon numbers showed a pronounced positive relationship with ALT levels, while LPCs, Cers, and SMs were considerably reduced in VPA-induced ALF subjects. Confirming this finding, markedly elevated levels of TAGs with higher summed carbons were also found in VPA-treated hepatic L02 cells. Moreover, we observed disrupted hepatic gene expressions involved in lipid metabolism and Akt–PPARγ pathway involved in the VPA-induced long-chain TAG accumulation. Our results demonstrate that large-scale lipidomics studies using ALF patients’ serum might lead to the identification of biomarkers of clinical diagnostic value for VPA-induced hepatotoxicity, and future studies are required to understand the mechanism of disruption in lipid homeostasis.

## Data Availability

The raw data supporting the conclusions of this manuscript will be made available by the authors, without undue reservation, to any qualified researcher.

## Ethics Statement

This study was approved by the ethics committee of Shengjing Hospital of China Medical University. Written informed consent was obtained from the guardians of each patient in accordance with the Declaration of Helsinki.

## Author Contributions

SX and LZ contributed to the study execution and manuscript preparation. YC, YM, TL, and MZ were responsible for the serum sample collection and lipidomics analysis. SX and YM contributed to the molecular biology experiments. ZW and LZ reviewed the manuscript. All authors approved the final version to be published.

## Funding

This project was supported by grants from the National Natural Science Foundation of China (Nos. 81673510, 81703628, and U1608282).

## Conflict of Interest Statement

TL was employed by Shanghai AB Sciex Analytical Instrument Trading Co. Ltd., China.

The remaining authors declare that the research was conducted in the absence of any commercial or financial relationships that could be construed as a potential conflict of interest.
