# Supplementary material for: Lipidomic Profiling Reveals Disruption of Lipid Metabolism in Valproic Acid-Induced Hepatotoxicity
Source: Front Pharmacol. 2019 Jul 19;10:819. doi: 10.3389/fphar.2019.00819 (PMC6659130; doi:10.3389/fphar.2019.00819)
Supplement: Supplementary file 1 [file DataSheet_1.pdf]

Supplementary Table S1 Specific primers used for quantitative PCR analysis

Name	Forward primer (5'to 3')	Reverse primer (5'to 3')
FATP1	ACAGGCACCTTCAAGATCCA	TGTGGCCAGTAGAGGAACAG
FATP2	CTGCCCTTTTACCACAGTGC	CGTAAGCCATTTCCCAGTGC
FATP3	TGGCAAATGAGGGCTTCGAC	CTGTACCGGGCAGTTGTGAG
FATP4	CCTCATCAACACCAACCTGC	TCTTTCAGCAGAGGGTCCAG
FATP5	CTTCCTCTACTTCCGCGACC	CCCACCTTACCCTCACAACC
FABP1	AGTTTCTCCGGCAAGTACCA	CCCCACCGTGAATTCGTTTT
CD36	CTTTGGCTTAATGAGACTGGGAC	GCAACAAACATCACCACACCA
ACC1	ATGTCTGGCTTGCACCTAGTA	CCCCAAAGCGAGTAACAAATTCT
FAS	CCCTCATCTCCCCACTCATC	CAGCGTCTTCCACACTATGC
ACOT1	GTGAGCTCTGCCTTTCCATG	GCTCTTCTGGTCAGGTCCTT
ACOT2	GTGAGCTCTGCCTTTCCATG	GCTCTTCTGGTCAGGTCCTT
SCD1	TCTAGCTCCTATACCACCACCA	TCGTCTCCAACTTATCTCCTCC
CPT1a	AACCCAGAGTACGTGTCCAG	TTTCAGGTGCCTTCCAAAGC
ALDH3A2	GAAGCAGCGATTTGACCACA	TTTCCCCAGGTTATGCGTCT
EHHADH	TGGATGTGGGCTGGAAATCT	TCTTCTGGCCAAATCGTCCT
AGPAT1	TCGTGCACTCTCTGGGATAC	GGTTCGTTCCTTTCCCCAAC
DGAT2	ATTGCTGGCTCATCGCTGT	GGGAAAGTAGTCTCGAAAGTAGC
ADPN	CCCATCTTTGTGCAGCTACC	ATAGGGCCACGAAACAGTCA
GPAM	GCACCCGAAACTGATAGCTG	CACTCACCCCATTCCTCACT
ABCB1	TGTTTTGTTCGGGACCACCA	GCCCTCCACTCATTTGAGCT
ABCG1	GTTTGTGACAGACTGCGTGT	CTTTGCCACTGATAGCGACC
ApoB100	TGAAAGACAACGTGCCCAAG	ATGGCCCCTTGATAAACCCA
ApoAI	TGTGTACGTGGATGTGCTCAAAG	GTCACGCTGTCCCAGTTGTCA
PPARA	CTTCGCAAACTTGGACCTGAA	GCTACCAGCATCCCGTCTTT
PPARG	GACCACTCCCACTCCTTTGA	GCAGGCTCCACTTTGATTGC
SREBP1c	TCAGCGAGGCGGCTTTGGAGCAG	CTGACCGACATCGAAGACATG
ChREBP	CGGGATGAGATTGAGGAGCT	ATGAGGATGCTGAACACCCA
HNF4a	CAACCCAACCTCATCCTCCT	CCACTCCAAGTTCCTGTTGC
CAR	TTCATCCATCACCAGCCCTT	CAGATTTCCACAGCTGCTCC
AhR	TTGAACCATCCCCATACCCC	TTCTGGCTGGCACTGATACA
PXR	AAGCCCAGTGTCAACGCAG	GGGTCTTCCGGGTGATCTC
SGMS1	TGAGCCTCTGGAGCATTTCA	CCGTTCTTGTGTGCTTCCAA
SGMS2	TCAGCCCAAGGTCAGAAGAAT	CTGGACTCAACCCGAACTTTC
KDSR	TACCCACCAGACACAGACAC	CCGAGAGCATGTACCCATCT
SPTLC1	AGGCCCTCAACATCATGGAA	CAGTCTGACATCTTGCTCGC
SPTLC2	GGGTTGTGAATGGGGAGGTA	CACTGAGCATCCACGTGAAG
SGPL1	CGAGATGACAAATGGGGCAG	GCGACCTATTGAAGTGCCTG
CERS2	TCTATATCACGCTGCCCCTG	CTTGCCACTGGTCAGGTAGA
CERS4	TCTCTGGTGCTGCTGTTACA	AGATGAGGAAGAGAGCGTCG
SMPD1	CTCTTCCTCACTGACCTGCA	CACCATATCAAAAGGGCCGG

Name	Forward primer (5'to 3')	Reverse primer (5'to 3')
LCAT	TGTGGGTGTGCTCTATGAGG	ATTGATGTGCTCCAGGGTCA
LPCAT1	TGAAGTGTGTTGCAGAACCG	ACAGAGACTCGAAACCAGGG
LPCAT2	ACAAGTCCCTCTGATTGGCA	TCCTCCTGATGTTGTTCGCT
LPCAT3	ACTCTCTCAGGGTCTCTCGT	GCAGCCTGTCTTTCCACAAT
LPCAT4	TCCCTCCATGACCCACTCTA	TCAGTCTCCCTTCTGCTTGG
LYPLA1	CGGTGTATGTGCGGCAATAA	GCCCAGTATCTCCCAATCCA
TNFα	AGGACCAGCTAAGAGGGAGA	CCCGGATCATGCTTTCAGTG
IL6	AGTCCTGATCCAGTTCCTGC	CTACATTTGCCGAAGAGCCC
TGFβ	AGACTTTTCCCCAGACCTCG	CAGAAGGTGGGTGGTCTTGA
GLUT1	TGTGCTCCTGGTTCTGTTCT	CAGCTCCTCGGGTGTCTTAT
GLUT2	GAGTTGGCGCTGTAAACATGG	GCACAAGTCCCACTGACATGAA
GLUT4	CCGCTACCTCTACATCATCCA	GCTTCCGCTTCTCATCCTTCA
GAPDH	ACACCCACTCCTCCACCTTT	CACCCTGTTGCTGTAGCCAA

Supplementary Table S2 Comprehensive list of identified 176 lipids from patient serum

Lipid name	Formula	Retention time (min)	Extract Mass (Da)	Mass Type
PC 30:0	C38H76O8NP	8.0	764.5436	$[M+CH3COOH-H]^-$
PC 32:0	C40H80O8NP	8.9	792.5749	$[M+CH3COOH-H]^-$
PC 32:1	C40H78O8NP	8.2	790.5593	$[M+CH3COOH-H]^-$
PC 32:2	C40H76O8NP	7.6	788.5436	$[M+CH3COOH-H]^-$
PC 34:1	C42H82O8NP	9.0	818.5906	$[M+CH3COOH-H]^-$
PC 34:2	C42H80O8NP	8.5	816.5749	$[M+CH3COOH-H]^-$
PC 34:3	C42H78O8NP	8.0	814.5593	$[M+CH3COOH-H]^-$
PC 34:4	C42H76O8NP	7.5	812.5436	$[M+CH3COOH-H]^-$
PC 36:2	C44H84O8NP	9.3	844.6062	$[M+CH3COOH-H]^-$
PC 36:3	C44H82O8NP	8.7	842.5906	$[M+CH3COOH-H]^-$
PC 36:4	C44H80O8NP	8.4	840.5749	$[M+CH3COOH-H]^-$
PC 36:5	C44H78O8NP	7.9	838.5593	$[M+CH3COOH-H]^-$
PC 36:6	C44H76O8NP	7.3	836.5436	$[M+CH3COOH-H]^-$
PC 38:2	C46H88O8NP	10.0	872.6375	$[M+CH3COOH-H]^-$
PC 38:3	C46H86O8NP	9.6	870.6219	$[M+CH3COOH-H]^-$
PC 38:4	C46H84O8NP	9.2	868.6062	$[M+CH3COOH-H]^-$
PC 38:5	C46H82O8NP	8.7	866.5906	$[M+CH3COOH-H]^-$
PC 40:4	C48H88O8NP	9.8	896.6375	$[M+CH3COOH-H]^-$
PC 40:5	C48H86O8NP	9.3	894.6219	$[M+CH3COOH-H]^-$
PC 40:6	C48H84O8NP	9.1	892.6062	$[M+CH3COOH-H]^-$
LPC 14:0	C22H46O7NP	2.0	526.3140	$[M+CH3COOH-H]^-$
LPC 16:0	C24H50O7NP	2.8	554.3453	$[M+CH3COOH-H]^-$
LPC 16:1	C24H48O7NP	2.3	552.3296	$[M+CH3COOH-H]^-$
LPC 18:0	C26H54O7NP	3.8	582.3766	$[M+CH3COOH-H]^-$
LPC 18:1	C26H52O7NP	3.0	580.3609	$[M+CH3COOH-H]^-$
LPC 18:2	C26H50O7NP	2.5	578.3453	$[M+CH3COOH-H]^-$
LPC 20:0	C28H58O7NP	4.8	610.4079	$[M+CH3COOH-H]^-$
LPC 20:3	C28H52O7NP	3.8	604.3609	$[M+CH3COOH-H]^-$
LPC 20:4	C28H50O7NP	2.5	602.3453	$[M+CH3COOH-H]^-$
PC O-32:1	C40H80O7NP	9.4	776.5800	$[M+CH3COOH-H]^-$
PC O-34:1	C42H84O7NP	9.6	804.6113	$[M+CH3COOH-H]^-$
PC O-34:2	C42H82O7NP	9.0	802.5957	$[M+CH3COOH-H]^-$
PC O-36:3	C44H84O7NP	9.2	828.6113	$[M+CH3COOH-H]^-$
PC O-36:4	C44H82O7NP	8.9	826.5957	$[M+CH3COOH-H]^-$
PC O-36:5	C44H80O7NP	8.8	824.5800	$[M+CH3COOH-H]^-$
PC O-36:6	C44H78O7NP	8.9	822.5644	$[M+CH3COOH-H]^-$
PC O-38:4	C46H86O7NP	9.8	854.6270	$[M+CH3COOH-H]^-$
PC O-38:5	C46H84O7NP	9.0	852.6113	$[M+CH3COOH-H]^-$

Lipid name	Formula	Retention time (min)	Extract Mass (Da)	Mass Type
PC O-38:6	C46H82O7NP	8.9	850.5957	$[M+CH3COOH-H]^-$
PC O-40:5	C48H88O7NP	9.8	880.6426	$[M+CH3COOH-H]^-$
PC O-40:6	C48H86O7NP	9.3	878.6270	$[M+CH3COOH-H]^-$
PE 34:2	C39H74O8NP	8.2	714.5079	$[M-H]^-$
PE 36:2	C41H78O8NP	8.0	742.5392	$[M-H]^-$
PE 36:4	C41H74O8NP	8.1	738.5079	$[M-H]^-$
LPE 16:0	C21H44O7NP	2.8	452.2783	$[M-H]^-$
LPE 18:0	C23H48O7NP	3.7	480.3096	$[M-H]^-$
LPE 18:1	C23H46O7NP	3.0	478.2939	$[M-H]^-$
LPE 18:2	C23H44O7NP	2.5	476.2783	$[M-H]^-$
LPE 20:4	C25H44O7NP	2.3	500.2783	$[M-H]^-$
LPE 22:6	C27H44O7NP	2.4	524.2783	$[M-H]^-$
PE O-34:3	C39H74O7NP	8.6	698.5130	$[M-H]^-$
PE O-36:5	C41H74O7NP	8.5	722.5130	$[M-H]^-$
PE O-38:5	C43H78O7NP	9.2	750.5443	$[M-H]^-$
PE O-38:6	C43H76O7NP	8.6	748.5287	$[M-H]^-$
PE O-40:6	C45H80O7NP	9.2	776.5600	$[M-H]^-$
PI 34:1	C43H81O13P	6.8	835.5342	$[M-H]^-$
PI 34:2	C43H79O13P	6.2	833.5186	$[M-H]^-$
PI 38:4	C47H83O13P	7.0	885.5499	$[M-H]^-$
SM 32:1;2	C37H75O6N2P	7.0	675.5436	$[M+H]^+$
SM 32:2;2	C37H73O6N2P	6.2	673.5279	$[M+H]^+$
SM 34:0;3	C39H81O7N2P	7.4	721.5854	$[M+H]^+$
SM 34:1;2	C39H79O6N2P	7.9	703.5749	$[M+H]^+$
SM 34:1;3	C39H79O7N2P	7.5	719.5698	$[M+H]^+$
SM 34:2;2	C39H77O6N2P	7.2	701.5592	$[M+H]^+$
SM 34:2;3	C39H77O7N2P	6.8	717.5541	$[M+H]^+$
SM 36:1;2	C41H83O6N2P	8.8	731.6062	$[M+H]^+$
SM 36:2;2	C41H81O6N2P	8.2	729.5905	$[M+H]^+$
SM 38:1;2	C43H87O6N2P	9.7	759.6375	$[M+H]^+$
SM 38:2;2	C43H85O6N2P	9.0	757.6218	$[M+H]^+$
SM 40:1;2	C45H91O6N2P	10.5	787.6688	$[M+H]^+$
SM 40:2;2	C45H89O6N2P	9.8	785.6531	$[M+H]^+$
SM 42:1;2	C45H95O6N2P	11.2	815.7001	$[M+H]^+$
SM 42:2;2	C45H93O6N2P	10.5	813.6844	$[M+H]^+$
SM 42:2;3	C45H93O7N2P	10.0	829.6793	$[M+H]^+$
Cer 34:1;2	C34H67N1O3	8.4	538.5194	$[M+H]^+$
Cer 36:1;2	C36H71N1O3	9.1	566.5507	$[M+H]^+$
Cer 40:1;2	C40H79N1O3	10.4	622.6133	$[M+H]^+$
Cer 42:1;2	C42H83N1O3	10.9	650.6446	$[M+H]^+$
Cer 42:2;2	C42H81N1O3	10.6	648.6289	$[M+H]^+$
Cer 44:1;2	C44H87N1O3	11.4	678.6759	$[M+H]^+$

Lipid name	Formula	Retention time (min)	Extract Mass (Da)	Mass Type
CE 16:0	C27H45C16H31O2	13.2	642.6184	$[M+NH4]^+$
CE 16:1	C27H45C16H29O2	12.9	640.6027	$[M+NH4]^+$
CE 18:1	C27H45C18H33O2	13.3	668.6340	$[M+NH4]^+$
CE 18:2	C27H45C18H31O2	13.0	666.6184	$[M+NH4]^+$
CE 18:3	C27H45C18H29O2	12.7	664.6027	$[M+NH4]^+$
CE 20:4	C27H45C20H31O2	12.9	690.6184	$[M+NH4]^+$
CE 20:5	C27H45C20H29O2	12.6	688.6027	$[M+NH4]^+$
CE 22:6	C27H45C22H31O2	12.7	714.6184	$[M+NH4]^+$
DAG 26:0	OHC29H55O4	7.5	502.4466	$[M+NH4]^+$
DAG 28:0	OHC31H59O4	8.3	530.4779	$[M+NH4]^+$
DAG 28:1	OHC31H57O4	7.7	528.4623	$[M+NH4]^+$
DAG 30:0	OHC33H63O4	9.0	558.5092	$[M+NH4]^+$
DAG 30:1	OHC33H61O4	8.6	556.4936	$[M+NH4]^+$
DAG 30:2	OHC33H59O4	8.0	554.4779	$[M+NH4]^+$
DAG 32:0	OHC35H67O4	9.8	586.5405	$[M+NH4]^+$
DAG 32:1	OHC35H65O4	9.3	584.5249	$[M+NH4]^+$
DAG 32:2	OHC35H63O4	8.8	582.5092	$[M+NH4]^+$
DAG 32:4	OHC35H59O4	9.3	578.4779	$[M+NH4]^+$
DAG 34:0	OHC37H71O4	10.4	614.5718	$[M+NH4]^+$
DAG 34:1	OHC37H69O4	9.9	612.5562	$[M+NH4]^+$
DAG 34:2	OHC37H67O4	9.4	610.5405	$[M+NH4]^+$
DAG 34:3	OHC37H65O4	8.9	608.5249	$[M+NH4]^+$
DAG 36:2	OHC39H71O4	10.0	638.5718	$[M+NH4]^+$
DAG 36:3	OHC39H69O4	9.5	636.5562	$[M+NH4]^+$
DAG 36:4	OHC39H67O4	9.1	634.5405	$[M+NH4]^+$
DAG 36:5	OHC39H65O4	8.7	632.5249	$[M+NH4]^+$
DAG 38:5	OHC41H69O4	9.5	660.5562	$[M+NH4]^+$
DAG 38:6	OHC41H67O4	9.0	658.5405	$[M+NH4]^+$
TAG 40:1	C43H80O6	11.4	710.6293	$[M+NH4]^+$
TAG 40:2	C43H78O6	11.1	708.6137	$[M+NH4]^+$
TAG 42:1	C45H84O6	11.8	738.6606	$[M+NH4]^+$
TAG 42:2	C45H82O6	11.5	736.6450	$[M+NH4]^+$
TAG 44:0	C47H90O6	12.5	768.7076	$[M+NH4]^+$
TAG 44:1	C47H88O6	12.2	766.6919	$[M+NH4]^+$
TAG 44:2	C47H86O6	11.9	764.6763	$[M+NH4]^+$
TAG 44:3	C47H84O6	11.5	762.6606	$[M+NH4]^+$
TAG 44:4	C47H82O6	11.3	760.6450	$[M+NH4]^+$
TAG 46:0	C49H94O6	12.9	796.7389	$[M+NH4]^+$
TAG 46:1	C49H92O6	12.6	794.7232	$[M+NH4]^+$
TAG 46:2	C49H90O6	12.3	792.7076	$[M+NH4]^+$
TAG 46:3	C49H88O6	12.1	790.6919	$[M+NH4]^+$
TAG 46:4	C49H86O6	11.8	788.6763	$[M+NH4]^+$

Lipid name	Formula	Retention time (min)	Extract Mass (Da)	Mass Type
TAG 46:5	C49H84O6	11.5	786.6606	$[M+NH4]^+$
TAG 48:0	C51H98O6	13.2	824.7702	$[M+NH4]^+$
TAG 48:1	C51H96O6	12.9	822.7545	$[M+NH4]^+$
TAG 48:2	C51H94O6	12.7	820.7389	$[M+NH4]^+$
TAG 48:3	C51H92O6	12.4	818.7232	$[M+NH4]^+$
TAG 48:4	C51H90O6	12.1	816.7076	$[M+NH4]^+$
TAG 48:5	C51H88O6	11.8	814.6919	$[M+NH4]^+$
TAG 50:1	C53H100O6	13.2	850.7858	$[M+NH4]^+$
TAG 50:2	C53H98O6	13.0	848.7702	$[M+NH4]^+$
TAG 50:3	C53H96O6	12.7	846.7545	$[M+NH4]^+$
TAG 50:4	C53H94O6	12.5	844.7389	$[M+NH4]^+$
TAG 50:5	C53H92O6	12.3	842.7232	$[M+NH4]^+$
TAG 50:6	C53H90O6	12.1	840.7076	$[M+NH4]^+$
TAG 52:2	C55H102O6	13.3	876.8015	$[M+NH4]^+$
TAG 52:3	C55H100O6	13.0	874.7858	$[M+NH4]^+$
TAG 52:4	C55H98O6	12.8	872.7702	$[M+NH4]^+$
TAG 52:5	C55H96O6	12.6	870.7545	$[M+NH4]^+$
TAG 52:6	C55H94O6	12.4	868.7389	$[M+NH4]^+$
TAG 52:7	C55H92O6	12.2	866.7232	$[M+NH4]^+$
TAG 52:8	C55H90O6	11.9	864.7076	$[M+NH4]^+$
TAG 54:3	C57H104O6	13.3	902.8171	$[M+NH4]^+$
TAG 54:4	C57H102O6	13.1	900.8015	$[M+NH4]^+$
TAG 54:5	C57H100O6	13.0	898.7858	$[M+NH4]^+$
TAG 54:6	C57H98O6	12.8	896.7702	$[M+NH4]^+$
TAG 54:7	C57H96O6	12.5	894.7545	$[M+NH4]^+$
TAG 54:8	C57H94O6	12.3	892.7389	$[M+NH4]^+$
TAG 56:1	C59H112O6	14.2	934.8797	$[M+NH4]^+$
TAG 56:2	C59H110O6	13.9	932.8641	$[M+NH4]^+$
TAG 56:3	C59H108O6	13.6	930.8484	$[M+NH4]^+$
TAG 56:4	C59H106O6	13.4	928.8328	$[M+NH4]^+$
TAG 56:5	C59H104O6	13.2	926.8171	$[M+NH4]^+$
TAG 56:6	C59H102O6	13.0	924.8015	$[M+NH4]^+$
TAG 56:7	C59H100O6	12.9	922.7858	$[M+NH4]^+$
TAG 56:8	C59H98O6	12.7	920.7702	$[M+NH4]^+$
TAG 56:9	C59H96O6	12.4	918.7545	$[M+NH4]^+$
TAG 58:4	C61H110O6	13.7	956.8641	$[M+NH4]^+$
TAG 58:5	C61H108O6	13.5	954.8484	$[M+NH4]^+$
TAG 58:6	C61H106O6	13.3	952.8328	$[M+NH4]^+$
TAG 58:7	C61H104O6	13.1	950.8171	$[M+NH4]^+$
TAG 58:8	C61H102O6	13.0	948.8015	$[M+NH4]^+$
TAG 58:9	C61H100O6	12.7	946.7858	$[M+NH4]^+$
TAG 60:8	C63H106O6	13.2	976.8328	$[M+NH4]^+$

Lipid name	Formula	Retention time (min)	Extract Mass (Da)	Mass Type
TAG 60:9	C63H104O6	13.0	974.8171	$[M+NH4]^+$
TAG 60:10	C63H102O6	12.9	972.8015	$[M+NH4]^+$
TAG 60:11	C63H100O6	12.7	970.7858	$[M+NH4]^+$
TAG 60:12	C63H98O6	12.5	968.7702	$[M+NH4]^+$
MAG 20:2	C23H42O4	2.8	400.3421	$[M+NH4]^+$
MAG 20:3	C23H40O4	2.2	398.3265	$[M+NH4]^+$
MAG 20:4	C23H38O4	1.7	396.3108	$[M+NH4]^+$
MAG 22:2	C25H46O4	3.7	428.3734	$[M+NH4]^+$
MAG 22:4	C25H42O4	2.4	424.3421	$[M+NH4]^+$
FFA 24:0	HC24H47O2	8.3	367.3582	$[M-H]^-$
FFA 24:1	HC24H45O2	7.3	365.3425	$[M-H]^-$
FFA 26:0	HC26H51O2	9.3	395.3895	$[M-H]^-$

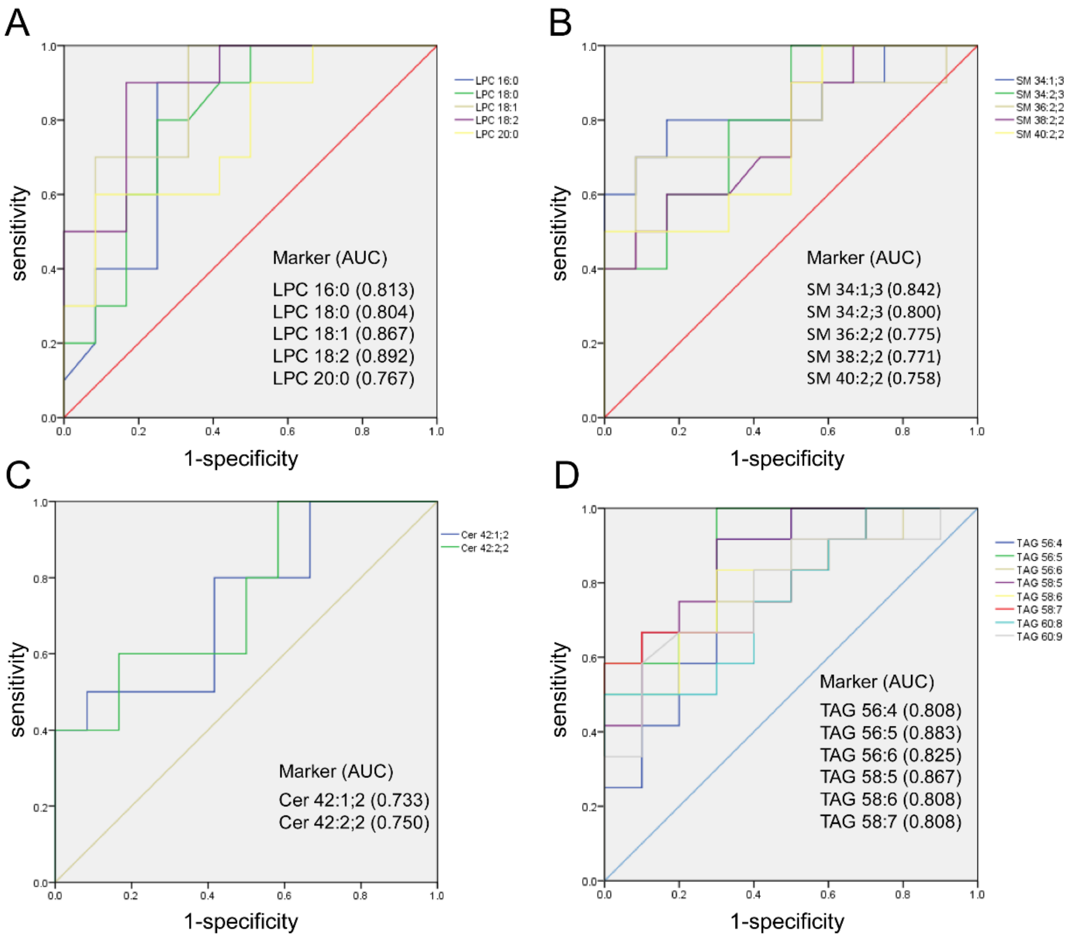

Supplementary Figure S1 ROC curves of the candidate biomarker for VPA hepatotoxicity diagnosis in epileptic children.

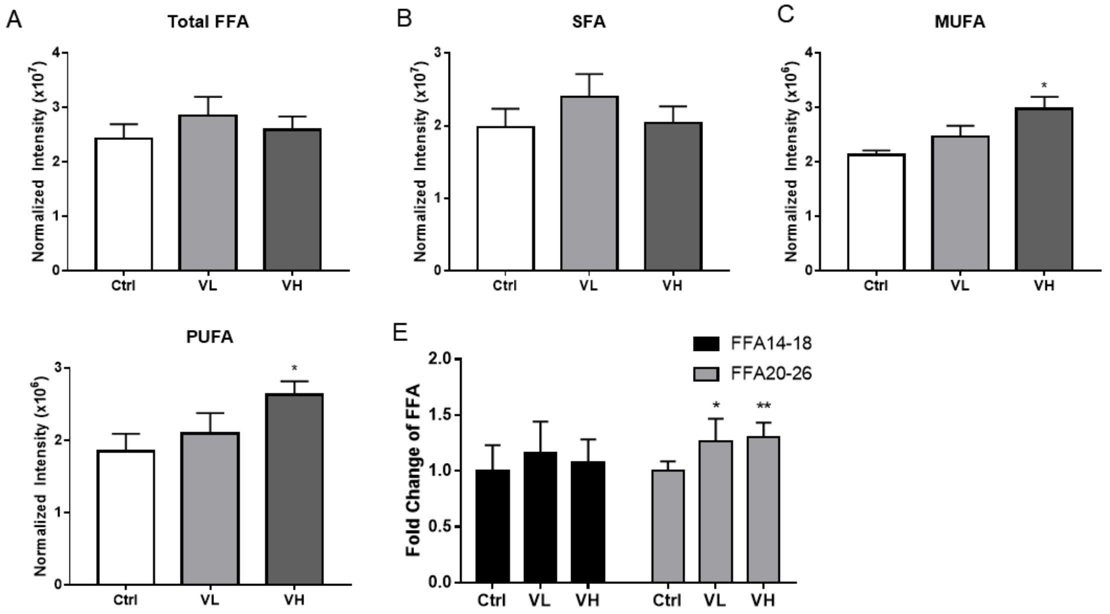

Supplementary Figure S2 Effect of VPA on FFA levels in L02 cells. Total hepatic FFA levels **(A)** and saturated fatty acid (SFA) **(B)** showed no difference among the groups. The hepatic monounsaturated fatty acid (MUFA) **(C)**, and polyunsaturated fatty acid (PUFA) levels **(D)** increased in VH (VPA 5 mM) group compared with control. Long-chain FFA (FFA20-26) increased in VL (VPA 1 mM) and VH (VPA 5mM) groups. Data were means ± SD, * $P < 0.05$ versus control group.

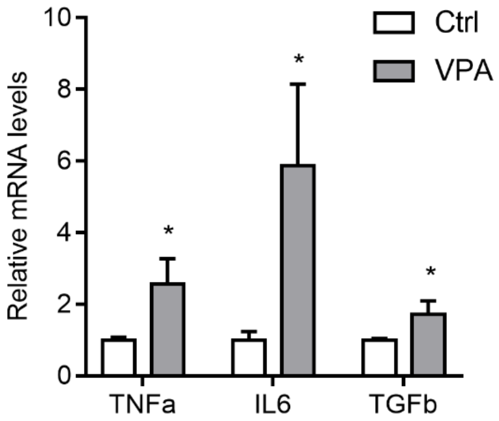

Supplementary Figure S3 VPA induced proinflammatory cytokines in hepatocytes. Real-time PCR analysis of *TNFα*, *IL6* and *TGFβ* mRNA expressions after VPA (1 mM) treatment for 24 h. The mRNA levels were normalized to those of GAPDH mRNA and subsequently normalized to those of control groups. Values were presented as mean ± SD, * $P < 0.05$ compared with control group ($n = 3$).

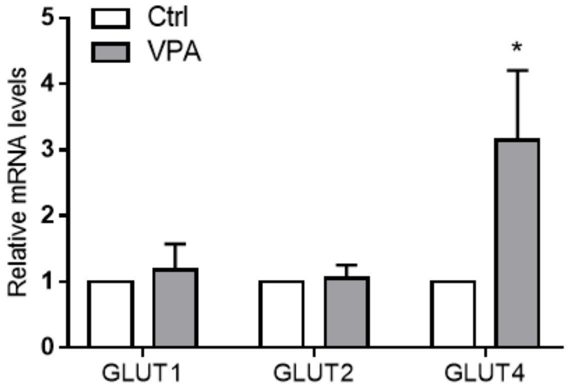

Supplementary Figure S4 Effect of VPA on mRNA levels of GLUTs in L02 cells. Real-time PCR analysis of GLUT1, GLUT2 and GLUT4 mRNA expressions after VPA (1 mM) treatment for 24 h. The mRNA levels were normalized to those of GAPDH mRNA and subsequently normalized to those of control groups. Values were presented as mean ± SD, * $P < 0.05$ compared with control group ($n = 3$).
